# Effect of red osier dogwood extract on growth performance, blood biochemical parameters, and gut functionality of broiler chickens challenged or unchallenged intraperitoneally with *Salmonella Enteritidis* lipopolysaccharide

**DOI:** 10.1016/j.psj.2022.101861

**Published:** 2022-03-18

**Authors:** Taiwo J. Erinle, Janice MacIsaac, Chengbo Yang, Deborah I. Adewole

**Affiliations:** ⁎ Department of Animal Science and Aquaculture, Faculty of Agriculture, Dalhousie University, Truro NS B2N 5E3, Canada; † Department of Animal Science, Faculty of Agricultural and Food Sciences, University of Manitoba, Winnipeg MB R3T 2N2, Canada

**Keywords:** red osier dogwood extract, broiler chickens, *Salmonella enteritidis* lipopolysaccharides, cecal microbiota, total antioxidant power

## Abstract

As we advance in the search for antibiotic-alternatives, harnessing plant materials with high total polyphenol concentration (TPC) would be quintessential. Given the high TPC in red osier dogwood (ROD) extract, the current study aimed to determine its efficacy on the growth performance, intestinal health, blood biochemistry, and antioxidant capacity of broiler chickens. A 21-day 4x2 factorial feeding trial was conducted based on two main factors namely, dietary treatments and *Salmonella Enteritidis* Lipopolysaccharides ***SE*-LPS**) challenge. A total of 384 one-day-old mixed-sex Cobb-500 broiler chicks were randomly allotted to four dietary treatments - Negative control (**NC**), NC + 0.05% bacitracin methylene disalicylate (**BMD**), NC + 0.3%**ROD**, and NC+0.5% **ROD**. Each treatment was assigned to eight replicates with six birds/replicate. On d 13 and 20, half of the birds were intraperitoneally injected with 1mL phosphate-buffered-saline /kg BW of birds (Unchallenged-group) and the remaining half with 1mg *SE*-LPS /kg BW of birds (Challenged-group). Average weight gain (**AWG**), average feed intake (**AFI**), feed conversion ratio (**FCR**), and mortality were determined weekly. On d 21, ten chickens/treatment were euthanized for measuring blood biochemical parameters, immune organ weights, caecal SCFA, and caeca microbiota. The SE-LPS decreased (*P* < 0.05) AWG and FCR on d 14 and 21, respectively. On d 14, 21, and overall basis, both ROD extract levels marginally improved (*P* < 0.05) the AWG of unchallenged birds compared to other treatments in the unchallenged-group. Challenged and unchallenged birds fed ROD extract had deeper (*P* < 0.05) crypt depth (CD) and higher villus height:CD, respectively, in the ileum. Globulin (GLB) and albumin:GLB were increased and reduced (*P* < 0.05), respectively, among birds fed 0.3%ROD compared to other treatments. There was no treatment effect on caeca SCFA, relative weight of immune organs, and serum antioxidants. Birds fed ROD extract had a higher (*P* < 0.05) relative abundance of caecal *Lactobacillus* and *Streptococcus* genera compared to the antibiotic treatment. Conclusively, incorporating 0.3% and 0.5%ROD extract into broiler chickens' nutrition improved growth performance and ileal morphology, and modified caecal microbiota of broiler chickens, regardless of the intraperitoneal *SE*-LPS challenge.

## INTRODUCTION

The global poultry industry is constantly embattled by the prevalence of disease-causing pathogens and their metabolites, undermining its performance, profitability, and survivability. On a global scale, the average annual economic burden these diseases pose on the poultry industry is $3 to $6 billion (Chapman and Jeffers, 2014). The current antibiotics restriction has consequentially contributed to the proliferation of intestinal pathogenic bacteria species that are known to impair chicken's health and cause food-borne infections for humans, including salmonellosis (Huyghebaert et al., 2011) and endotoxaemia (Acharya and Bajaj, 2017). Both disease conditions are often caused by pathogenic *Salmonella* or their lipopolysaccharides. While the liveability and growth improvement potentials of antibiotic drugs on poultry species cannot be contested, the iron-fisted restriction to their use is quite understandable and would further prevent the menace of antibiotic-resistant bacteria resulting from the overwhelming use of critical and high priority antibiotics. There have been continuous efforts directed at identifying suitable alternatives to antibiotic use in poultry production. Suitable antibiotic alternatives could be referred to as feed additives, such as plant extracts, beneficial microbial culture, special plant fiber, antimicrobials, or other metabolites usually of natural origin that could particularly improve growth and gut health, in a marginal, equivalent and/or better capacity compared to antibiotic effect given the same condition.

Of the ubiquitous bacteria species, the gram-negative ones are the most economically significant group recognized for their appreciable resistance to some antimicrobials due to the extra unique structure in their outer membrane. The implicated bacteria in this category, include *Salmonella* spp, which produces lipopolysaccharides (**LPS**; an endotoxin containing virulence factors and proteins), contributing to the structural integrity of the bacteria. Interestingly, LPS comprises O antigen, oligosaccharides, and lipid A, thus, accounting for about 75 to 80% of the outer membrane of gram-negative bacteria (Klein and Raina, 2019; Avila-Calderón et al., 2021). Lipopolysaccharides are powerful immune-stimulants that trigger the innate immune response of the host (Klein and Raina, 2019; Valdez, 2021) upon recognition by Toll-like receptors in cells, including monocytes, beta cells, and macrophages, thus promoting the secretion of proinflammatory cytokines. The gastrointestinal tract hosts trillions of gram-positive or gram-negative bacteria. Lipopolysaccharides have been allegedly reported to be present sometimes in a healthy gut (Reisinger et al., 2020); however, at a certain threshold, they cause inflammation, fever, diarrhea, septic shock, and potential death (Wassenaar and Zimmermann, 2018; Farhana, 2021) provided they are from pathogenic bacteria like *Salmonella*. The secretion of LPS is not limited to the host's gut, as a reasonable amount of LPS concentration could also be detected in food/feed (Wassenaar and Zimmermann, 2018). In broiler chicken studies, LPS have been implicated in the impairment of performance characteristics (Zheng et al., 2016; Chen et al., 2020; Zhang et al., 2020), inducement of oxidative stress (Li et al., 2015; Zhang et al., 2020), causation of intestinal inflammation (Zhang et al., 2020) and disruption of the structural integrity of the intestinal wall, gut barrier functions and nutrient absorption (Zhang et al., 2020). Farhana (2021) reported that LPS is often used in serotyping gram-negative bacteria, and their early detection in the serum could be a diagnostic marker for infection. Thus, the severity of LPS on the host's immune system is expected to vary depending on the chemical structure of LPS and the bacteria-type producing it. Responses of broiler chickens to LPS derived from *E. coli* and *Salmonella* typhimurium are the most reported in research. However, there is a paucity of ample information on broiler chickens’ response when challenged with Salmonella Enteritidis LPS.

Remarkably, some studies have demonstrated that oxidative stress caused by LPS could be improved by dietary supplementation of exogenous antioxidants (Wu et al., 2013; Jang et al., 2014). Interestingly, bioactive substances in plants have been well-documented to possess antioxidant prowess in an equivalent capacity of some vitamins, such as vitamin E (Mazur-Kuśnirek et al., 2019). An example of such plants with a high concentration of bioactive substances is the red osier dogwood (**ROD**) plant*.*

Red osier dogwood (*Cornus stolonifera*) is a naturally growing ornamental shrub in all provincial areas of Canada (Scales, 2015). Red osier dogwood is a phytogenic additive that has been reported to contain a high concentration of phenolic compounds, primarily gallic acids, quercetin, rutin, and anthocyanins (Isaak et al., 2013; Scales, 2015). Dietary Supplementation of ROD reduced antibiotics usage in weaned pigs and limited the occurrence of diarrhea and death in rabbits (Scales, 2015); it afforded protection to weanling pigs against oxidative stress induced by *E. coli* infection (Amarakoon, 2017), and maintained growth performance and improved nutrient digestibility and absorption, and livability in broiler chickens (Mogire et al., 2021). In fact, in vitro demonstrations mimicking a real-time intestinal absorption of ROD extract bioactive substances using Caco-2 cells confirmed that the phenolic compounds in ROD are capable of mitigating inflammatory responses by stepping down the production of interleukin-8 secretion and reactive oxygen species and stepping up the production of body antioxidant enzymes including superoxide dismutase and glutathione peroxidase (Jiang et al., 2019; Yang et al., 2019). A considerable number of studies involving dietary application of ROD extract have been demonstrated on swine, rabbits, equine, bovine, and poultry. The study conducted by Mogire et al. (2021) was the first to investigate the use of ROD at 0.1 and 0.3% inclusion rate in broiler chickens but lacked an immune-response challenge model. To the best of our knowledge, there is currently no study on the effect of ROD extract on blood parameters, antioxidant, and gut health of broiler chickens challenged with LPS. Thus, there is a need for more research to establish the antioxidant and antimicrobial activities of ROD extract in broiler chickens with stressed immune system.

Given the improved gut health and maintained growth performance in broiler chickens fed 0.1 and 0.3% dietary ROD extract, we hypothesized that a higher inclusion level of 0.3 and 0.5% might be better treatment combinations that will attenuate the triggered immune response of broiler chickens challenged intraperitoneally with *SE*-LPS, without compromising their growth performance. Thus, this study aimed to examine the ameliorative potential of dietary ROD extract at 0.3 and 0.5% as an alternative to in-feed antibiotics on growth performance, blood biochemical parameters, gut health, and antioxidant status of broiler chickens challenged intraperitoneally with *SE*-LPS.

## MATERIALS AND METHODS

The experimental protocol was approved by Dalhousie University Animal Care and Use Committee (Animal Care Certification Number 2020-043). The birds were handled following the guidelines established by the Canadian Council on Animal Care (2009).

### Birds and Housing

A total of 384 one-day-old mixed-sex Cobb-500 broiler chicks were obtained from Atlantic Poultry Incorporated, Port Williams, Nova Scotia, and were raised in a 2-tier battery cage system (0.93 m × 2.14 m) at a stocking density of 0.076 m^2^/birds for 21 d. Upon arrival, the mixed-sex birds were weighed in groups of 6 and randomly allocated to each cage. The room temperature was monitored daily and was gradually reduced from 32 to 24°C from d 0 to 21. The lighting program was set to produce 18 h of light and 6 h of darkness throughout the experimental period, and illumination was gradually reduced from 20 1 × on d 0 to 5 1 × on d 21.

### Diets and Experimental Design

**Figure 1:**
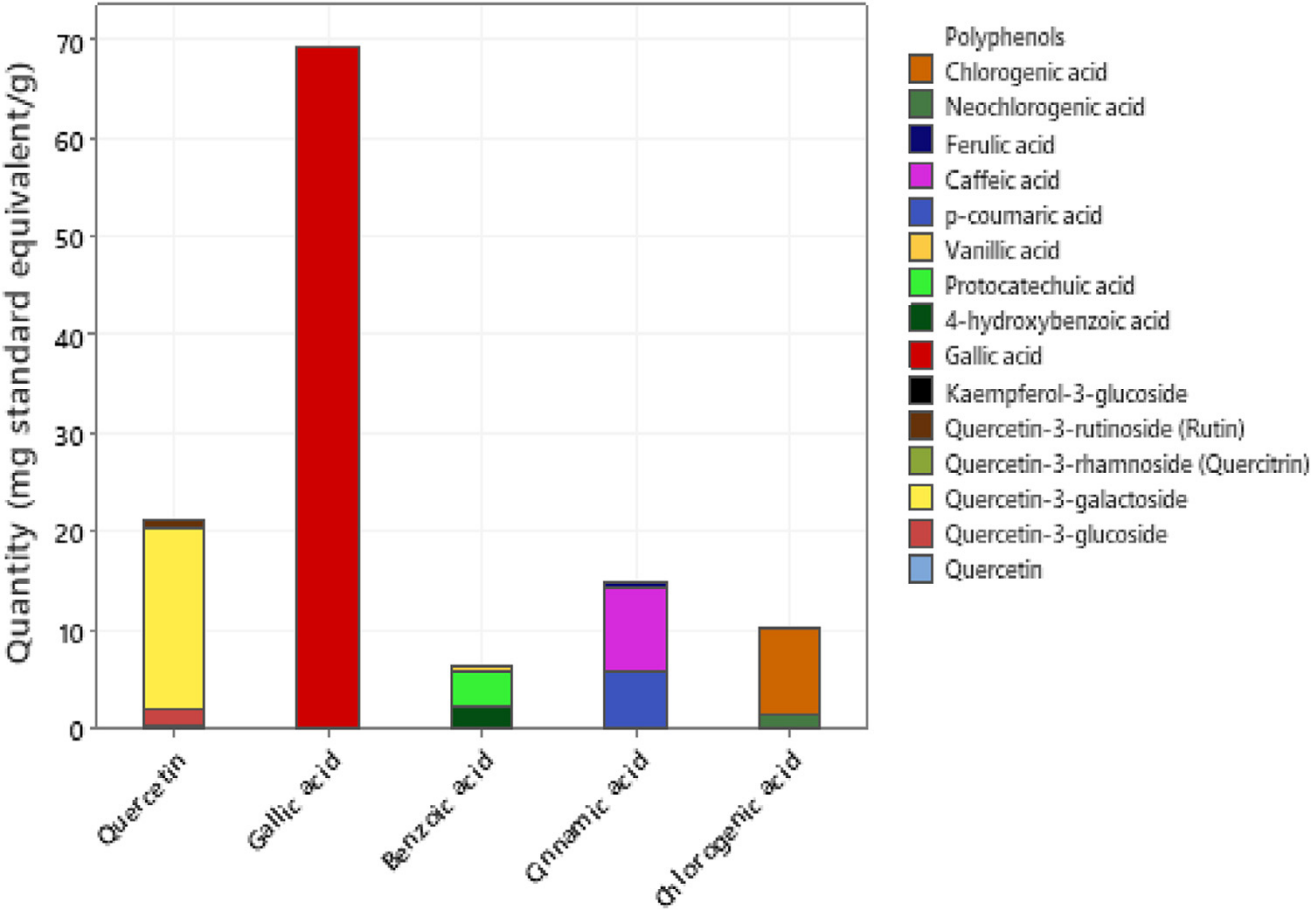
Polyphenols profile of red osier dogwood extract by UPLC-MSMS (mg standard equivalent/g).

The ROD extract used in this study was obtained from Red Dogwood Enterprise, MB, Canada. The birds were randomly assigned to 8 treatments groups containing eight replicate cages of 6 birds each. The experiment was designed as a 4 × 2 factorial arrangement based on 2 main factors, as shown in Table 1. The main factors were: 1) 4 dietary treatments: corn-wheat-soybean meal based diet negative control (**NC**), NC with 0.05% bacitracin methylene disalicylate (**BMD**) per kilogram of diet; and NC supplemented with 0.3 or 0.5% ROD extract and 2) two intraperitoneal injections: 1 mL sterile 1 × phosphate buffered saline (**PBS**) per kg BW of birds (AVL82762, HyClone Laboratories, Inc., Logan, UT) as the unchallenged group (**U**), or 1 mg SE-LPS per kg BW of birds (ATCC 13076; Sigma-Aldrich, St. Louis, MO) as the challenged group (**C**). The intraperitoneal injection was carried out on d 13 and 20. The basal diet was formulated as isocaloric and isonitrogenous to meet the nutrient requirements of broiler chickens as recommended by National Research Council (1994). The compositions of the experimental diets are presented in Table 2. Experimental diets containing BMD and ROD were mixed from a single basal diet, thus, the reason for reporting analyzed nutrient contents of the basal diets only. The proximate composition of the control diets was determined following AOAC (1990) procedure. The phenolic profiling of ROD extract used in our study is presented in Figure 1. Total polyphenols in the ROD and diets at the starter and grower phases and polyphenols profile of ROD (Folin-Coicalteu) were determined using ultra-performance liquid chromatography-tandem mass spectrometer (**UPLC-MS/MS**) at the Institute of Nutrition and Functional Foods, Quebec, Canada.

**Table 1:** Experimental design.

	Challenge model	
Dietary treatment (number of replicates; n)	Unchallenged (U)	Challenged (C)
- Basal (NC)	(1) NC + PBS (n = 8)	(2) NC + *SE*-LPS (n = 8)
+ Antibiotic	(3) NC + BMD + PBS (n = 8)	(4) NC + BMD + *SE*-LPS (n = 8)
+ 0.3% ROD extract	(5) NC + 0.3% ROD + PBS (n = 8)	(6) NC + 0.3% ROD + *SE*-LPS (n = 8)
+0.5% ROD extract	(7) NC + 0.5% ROD + PBS (n = 8)	(8) NC + 0.5% ROD + *SE*-LPS (n = 8)

NC = Basal diet or negative control, BMD = bacitracin methylene disalicylate antibiotic, 0.3% ROD = diet containing 0.3% red osier dogwood extract, 0.5% ROD = diet containing 0.5% red osier dogwood extract.

C, Challenged group; PBS, Intraperitoneal injection of phosphate buffered saline; *SE*-LPS, Intraperitoneal injection of Salmonella Enteritidis lipopolysaccharide; U, Unchallenged group.

**Table 2:** Gross and nutrient compositions of experimental diets (as-fed basis, %, unless otherwise stated)^1^.

Ingredients	Starter phase (1–14 d)				Grower phase (14–21 d)			
	Basal	BMD	0.3% ROD	0.5% ROD	Basal	BMD	0.3% ROD	0.5% ROD
Corn	42.37	42.27	41.83	41.48	45.99	45.65	45.22	44.86
Soybean meal (47.5%CP)	40.13	40.15	40.17	40.2	36.15	36.21	36.24	36.26
Wheat	10.00	10.00	10.00	10.00	10.00	10.00	10.00	10.00
Vegetable oil	2.82	2.85	3.01	3.14	3.74	3.85	4.01	4.14
Dicalcium phosphate	1.57	1.57	1.57	1.57	1.39	1.39	1.39	1.39
Limestone	1.45	1.45	1.45	1.45	1.32	1.32	1.32	1.32
DL Methionine premix^W^	0.61	0.61	0.61	0.61	0.53	0.53	0.53	0.53
Starter Vitamin/Mineral premix^X^	0.50	0.50	0.50	0.50	-	-	-	-
Grower/Finisher Vitamin/Mineral premix^Y^	-	-	-	-	0.50	0.50	0.50	0.50
Sodium chloride	0.40	0.40	0.40	0.40	0.38	0.38	0.38	0.38
Red dogwood extract	-	-	0.30	0.50	-	-	0.30	0.50
BMD 110 G Z	-	0.05	-	-	-	0.05	-	-
Lysine HCL	0.16	0.16	0.16	0.16	-	0.12	0.12	0.12
Formulated composition								
Crude protein	23	23	23	23	21.5	21.5	21.5	21.5
Metabolizable energy (kcal kg^−1^)	3,000	3,000	3,000	3,000	3,100	3,100	3,100	3,100
Calcium	0.96	0.96	0.96	0.96	0.87	0.87	0.87	0.87
Available phosphorus	0.48	0.48	0.48	0.48	0.44	0.44	0.44	0.44
Digestible lysine	1.28	1.28	1.28	1.28	1.15	1.15	1.15	1.15
Digestible Methionine + Cystine	0.95	0.95	0.95	0.95	0.87	0.87	0.87	0.87
Sodium	0.19	0.19	0.19	0.19	0.18	0.18	0.18	0.18
Analyzed composition								
Crude protein	24.1				22.1			
Calcium	0.81				0.75			
Total phosphorus	0.68				0.62			
Sodium	0.15				0.12			
Crude fat	3.22				4.40			

W Supplied/kg premix: DL-Methionine, 0.5 kg; wheat middlings, 0.5 kg.

X Starter vitamin-mineral premix contained the following per kg of diet: 9,750 IU vitamin A; 2,000 IU vitamin D3; 25 IU vitamin E; 2.97 mg vitamin K; 7.6 mg riboflavin; 13.5 mg Dl Ca-pantothenate; 0.012 mg vitamin B12; 29.7 mg niacin; 1.0 mg folic acid, 801 mg choline;0. 3 mg biotin; 4.9 mg pyridoxine; 2.9 mg thiamine; 70.2 mg manganese; 80.0 mg zinc; 25 mg copper; 0.15 mg selenium; 50 mg ethoxyquin; 1543mg wheat middlings; 500 mg ground limestone.

Y Grower and Finisher vitamin-mineral premix contained the following per kg of diet: 9,750 IU vitamin A; 2,000 IU vitamin D3; 25 IU vitamin E; 2.97 mg vitamin K; 7.6 mg riboflavin; 13.5 mg Dl Ca-pantothenate; 0.012 mg vitamin B12; 29.7 mg niacin; 1.0 mg folic acid, 801 mg choline;0. 3 mg biotin; 4.9 mg pyridoxine; 2.9 mg thiamine; 70.2 mg manganese; 80.0 mg zinc; 25 mg copper; 0.15 mg selenium; 50 mg ethoxyquin; 1,543 mg wheat middlings; 500 mg ground limestone.

Z Bacitracin methylene disalicylate (providing 55 mg/kg mixed feed); Alpharma, Inc., Fort Lee, NJ.

1 Basal, negative control diet, BMD (bacitracin methylene disalicylate) antibiotic diet, 0.3% ROD, diet containing 0.3% red osier dogwood extract, 0.5% ROD, diet containing 0.5% red osier dogwood extract.

### Growth Performance and Sample Collection

Average body weight (**ABW**) and average feed intake (**AFI**) were determined weekly on a cage basis, and mortality was recorded daily to correct for AFI and feed conversion ratio (**FCR**). Birds that died were sent to the Veterinary Pathology Laboratory, Dalhousie University for postmortem examinations.

### Blood Biochemistry and Antioxidant Assay

On d 21, eighty birds (10 birds from each treatment) were randomly selected, individually weighed, and euthanized by electrical stunning and exsanguination. At exsanguination, approximately 8 mL of blood samples were collected from the jugular vein of each bird and were divided into 2 aliquots (4 mL each) in 5 mL heparinized tube and 5 mL serum tube for the determination of plasma biochemistry, and serum enzyme-link immunosorbent assay (**ELISA**), respectively. Samples for blood biochemical analysis were centrifuged at 5,000 rpm for 10 m and shipped on ice to Atlantic Veterinary College, University of Prince Edward Island Pathology Laboratory, where samples were analyzed using Cobas 6000 analyzer series. Serum immunoglobulin Y (**IgY**) and immunoglobulin M (**IgM**) were analyzed using respective ELISA kits from Bethyl Laboratories, Inc. (catalog number E33-104-200218 and E33-102-180410) following manufacturer instructions. Superoxide dismutase (**SOD**) and total antioxidant power (**TAP**) were analyzed using superoxide dismutase assay kit (Item Number 706002; Cayman Chemical, Ann Arbor, MI) and Oxiselect total antioxidant capacity assay kit (MAK187-1KT; Sigma-Aldrich), respectively following the manufacturers’ instructions.

### Gut Morphology

A 1.5 cm segment at the middle of the duodenum jejunum, and ileum were collected and preserved in 10% buffered formalin (Sigma-Aldrich) for 3 d. The formalin-preserved intestinal segments were immersed in paraffin and cross-sectioned. Each of the cross-sectioned segments was mounted on a glass slide (n = 10 per treatment) and stained with Alcian blue and periodic acid-Schiff (**PAS**) reagents. The morphological slides were examined under a microscope coupled with a digital camera. Ten well-oriented and distinct villi on each slide were identified and measured for villus height (**VH**), villus width (**VW**), and crypt depth (**CD**). Villus height was measured from the tip of the villus to the villus crypt junction, that is, top of the lamina propria of each villus. Crypt depth was measured from the villus crypt junction to the tip of the muscularis mucosa (Shang et al., 2020). The villus height:crypt depth ratio (**VH:CD)** was subsequently estimated.

### Short-Chain Fatty Acid Concentrations and Total Eubacteria Count

Digesta from the pair of ceca were mixed and divided into 2 subsamples. The cecal samples for SCFA and total eubacteria determination were immediately preserved using BioFreeze sampling kits (Alimetrics Diagnostics Ltd., Espoo, Finland) following the manufacturer's recommended protocol. In addition to the cecal SCFA concentration, the analysis of the most prevalent bacterial species was performed by Alimetrics Diagnostics Ltd.

### Gut Microbiota

The second portion of the mixed cecal digesta were stored in plastic RNAse and DNAse-free tubes, placed in liquid nitrogen, and followed by storage at −80°C for further gut microbiota analysis. Specimens were placed into a MoBio PowerMag Soil DNA Isolation Bead Plate (Qiagen, Carlsbad, CA). DNA was extracted following MoBio's instructions on a KingFisher robot. Bacterial 16S rRNA genes were PCR-amplified with dual-barcoded primers targeting the V4–V5 region (515FB 5’-GTGYCAGCMGCCGCGGTAA-3’ and 926R 3’ CCGYCAATTYMTTTRAGTTT-5’). Amplicons were sequenced with an Illumina MiSeq using the 300-bp paired-end kit (v.3) at the Integrated Microbiome Resource (http://imr.bio) of Dalhousie University. Sequences were denoised, taxonomically classified using Greengenes (v. 13_8) as the reference database, and clustered into 97%-similarity operational taxonomic units (**OTU**) with the mothur software package (v. 1.39.5) (Schloss, 2009), following the recommended procedure (https://www.mothur.org/wiki /MiSeq_ SOP; accessed Nov 2017). Bioinformatics analyses were conducted in the R statistical environment (R Development Core Team, 2013).

### Relative Weight of Immune Organs

Two lymphoid organs (spleen and liver) were collected from each bird, and the relative weight was expressed as a percentage to the individual BW.

### Statistical Analysis

Datasets were subjected to 4 × 2 factorial analysis of variance (**ANOVA**) using General Linear Model of Minitab LLC, (2019) software. Error terms of individual response variable were confirmed for the validity of 3 basic assumptions including, normality, constant variance, and independence. Normal probability plot of residuals was done to verify the normality of error terms using the Anderson Darling test in the same statistical package. Where error terms of datasets were found to be non-normal or non-constant, the respective original datasets were subjected to various transformation functions. If upon transformations, normality and homoscedasticity of the error terms were still violated, then such datasets were analyzed using the Kruskal-Wallis test. Following ANOVA, differences between significant means were separated using Tukey's honest significant difference (**HSD**) test and Mann Whitney for the parametric and nonparametric dataset, respectively in the same statistical package. Analyzed datasets were presented as means, standard error of the mean (**SEM**), and probability values. Statistically different values were considered at *P* < 0.05.

## RESULTS

### Total Polyphenol Content

**Figure 2:**
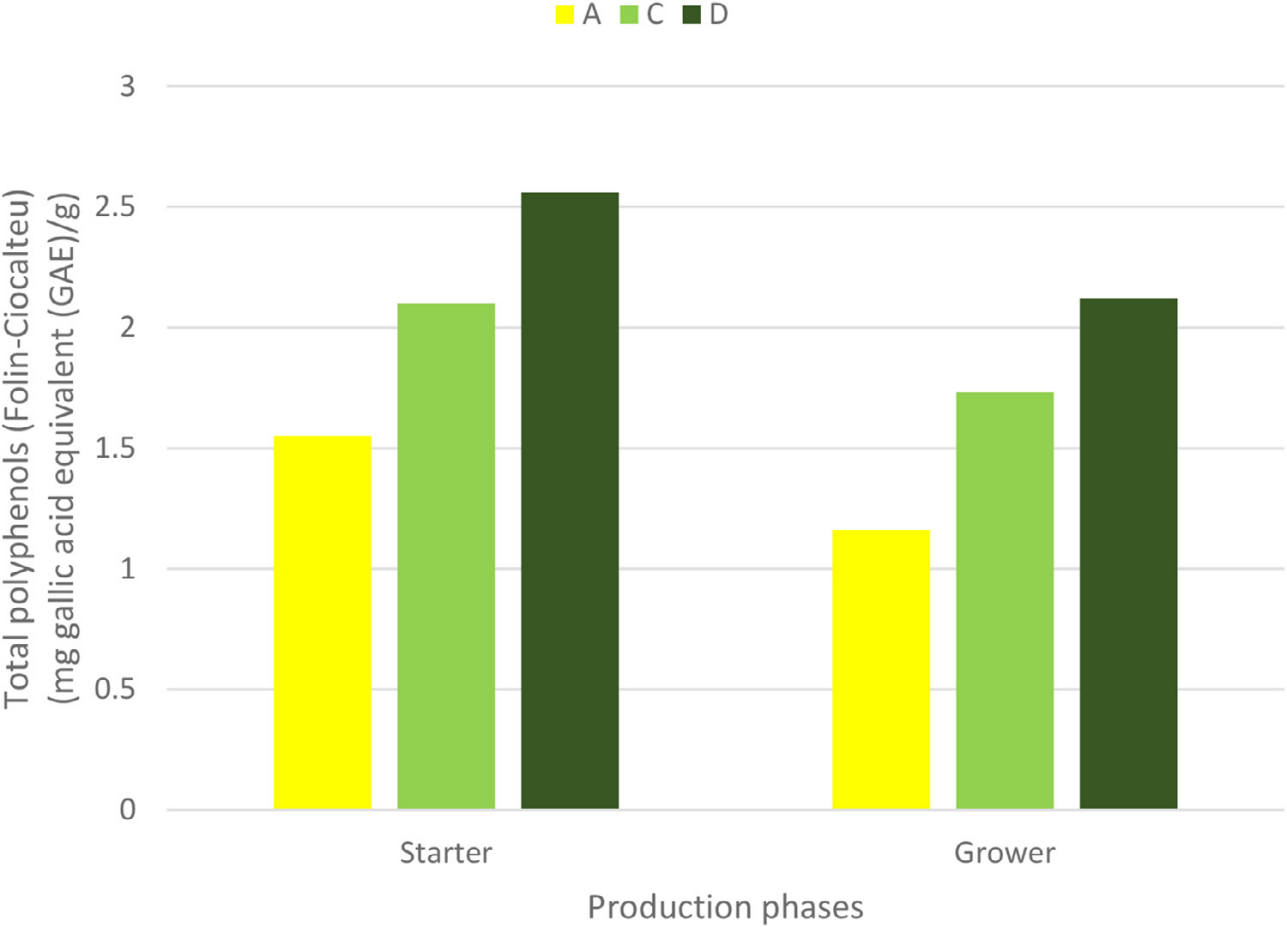
Total polyphenols content (mg gallic acid equivalent GAE/g) in treatments fed to broiler chicken according to production phases. A, B, C, and D diets per production phase. Treatment: A = Negative control, C = diet containing 0.3% red osier dogwood extract, and D = diet containing 0.5% red osier dogwood extract.

The result of the polyphenol profile of ROD extract (mg standard equivalent/g) and the TPC of the dietary treatments are presented in Figures 1 and 2, respectively. The measured total polyphenol in the ROD extract was 238.81 mg gallic acid equivalent (**GAE**)/g. From the polyphenol profile of ROD extract, gallic acid and quercetin were observed to be most abundant phenolic compounds. The TPC (Folin-Ciocalteu) (mg GAE/g) in the starter diets namely, A, C, and D, respectively were 1.55, 2.1, and 2.56, respectively. However, in the grower phase, TPC in A, C, and D were 1.16, 1.73, and 2.12 mg GAE/g.

### Growth Performance

The effects of ROD extract as antibiotic-alternative on growth performance of broiler chickens challenged or unchallenged with *SE*-LPS is presented in Table 3. No interaction effect was observed; thus, the results are reported based on the main effects. No treatment effects (*P* > 0.05) was observed on the growth response of the unchallenged birds on d 7 and the challenged birds throughout the entire experimental period. However, on d 14 and d 21, and overall basis, AWG of unchallenged group of birds followed a specific pattern of variation and was observed to be marginally improved (*P* < 0.05) among birds fed dietary 0.3 and 0.5% ROD extract compared with those fed antibiotic and control diets. On d 14, there was a significant model effect (*P* < 0.05) on AWG and was seen to be significantly higher (*P* < 0.05) among the unchallenged group of birds compared to the challenged birds. On d 21, there was a significant treatment × challenge model interaction effect (*P* < 0.05) on AFI of birds. Meanwhile, the FCR was significantly influenced (*P* < 0.05) by the dietary challenge model and was observed to be lower among the unchallenged birds compared with the challenged birds. The dietary treatments and model did not affect mortality.

**Table 3:** Effect of red osier dogwood extract on growth performance of broiler chickens challenged intraperitoneally with *Salmonella Enteritidis* Lipopolysaccharide examined at weekly levels.

			Treatment^1^					Challenge model^2^			P-Value		
Days	Parameters	Challenge model^2^	Basal	BMD	0.3% ROD	0.5% ROD	SE Mean^3^	U	C	SE Mean^3^	Treatment Effect	Model Effect	Interaction Effect
	Average feed intake (g/bird)	U	128.2	116.5	118.5	119.2	2.110	120.0	123.7	1.450	0.217	0.189	0.226
		C	125.7	128.3	120.5	119.2	1.980						
Day 0–7	Average weight gain (g/bird)	U	97.3	98.8	95.4	91.1	1.920	95.7	98.0	1.300	0.612	0.379	0.835
		C	98.6	99.3	96.4	97.7	1.750						
	FCR	U	1.33	1.19	1.27	1.31	0.031	1.27	1.28	0.020	0.828	0.931	0.287
		C	1.29	1.33	1.26	1.23	0.030						
	Mortality(%)	U	0.00	0.00	0.00	0.00	0.080	0.00	0.00	0.060	0.822	1.000	-
		C	0.00	0.00	0.00	0.00	0.080						
	Average feed intake (g/bird)	U	287.5	328.6	303.9	300.8	6.500	304.1	294.1	4.300	0.127	0.240	0.481
		C	284.3	299.6	284.8	305.1	5.600						
Day 7–14	Average weight gain (g/bird)	U	208.7^b^	250.9^a^	243.3^ab^	228.0^ab^	5.580	234.8^a^	222.9^b^	3.330	0.013	0.048	0.197
		C	219.0	234.5	215.9	218.9	3.480						
	FCR	U	1.38	1.31	1.24	1.31	0.022	1.30	1.32	0.020	0.261	0.695	0.076
		C	1.30	1.28	1.32	1.40	0.022						
	Mortality(%)	U	0.00	0.00	0.00	0.00	0.040	0.00	0.00	0.030	0.552	1.000	-
		C	0.00	0.00	0.00	0.00	0.040						
	Average feed intake (g/bird)	U	680.5	723.4	750.4	694.2	9.360	712.1	717.3	7.190	0.208	0.710	0.047
		C	748.4	725.3	708.1	687.2	11.10						
Day 14–21	Average weight gain (g/bird)	U	346.6^b^	416.0^a^	403.9^ab^	378.7^ab^	8.680	386.3	367.0	5.630	0.019	0.063	0.081
		C	372.6	387.9	358.6	349.1	6.880						
	FCR	U	2.00	1.75	1.87	1.84	0.040	1.86^b^	1.96^a^	0.030	0.045	0.036	0.818
		C	2.02	1.88	1.99	1.98	0.030						
	Mortality(%)	U	0.00	0.00	0.00	0.00	0.030	0.00	0.00	0.020	0.566	1.000	
		C	0.00	0.00	0.00	0.00	0.030						
	Average feed intake (g/bird)	U	1,096	1,169	1,173	1,114	11.80	1,138	1,136	8.420	0.201	0.923	0.069
		C	1,162	1,155	1,115	1,114	12.20						
Overall (Day 0–21)	Average weight gain (g/bird)	U	652.6^b^	765.7^a^	742.6^ab^	697.9^ab^	15.10	714.7	687.1	9.210	0.018	0.108	0.140
		C	690.2	721.7	671.0	665.7	10.30						
	FCR	U	1.70	1.53	1.59	1.60	0.030	1.61	1.66	0.016	0.053	0.096	0.716
		C	1.69	1.61	1.67	1.68	0.020						
	Mortality (%)	U	0.00	0.00	0.00	0.00	0.030	0.00	0.00	0.019	0.422	0.793	-
		C	0.00	0.00	0.00	0.00	0.025						

^ab^In a row, means assigned different lowercase letters are significantly different, *P* < 0.05 (Tukey's procedure).

1 Basal, negative control diet, BMD (bacitracin methylene disalicylate) antibiotic diet, 0.3% ROD, diet containing 0.3% red osier dogwood extract, 0.5% ROD, diet containing 0.5% red osier dogwood extract.

2 U = Unchallenged group; C = Challenged group.

3 SEM = standard error of the mean.

### Gut Morphology

The effects of ROD extract on the intestinal morphology of broiler chickens challenged or unchallenged intraperitoneally with *SE*-LPS is presented in Table 4. There was no interaction and model effects; therefore, the results were interpreted based on the treatment effects. With the exception of ileal CD and VH:CD, dietary supplementation of 0.3 and 0.5% ROD extract did not have significant effect on gut morphology variables of the broiler chickens. In the ileal section, both levels of ROD extract significantly deepened (*P* < 0.05) CD among the challenged birds compared with the antibiotic and control birds. Dietary supplementation of 0.3% ROD extract significantly improved (*P* < 0.05) VH:CD compared with other treatments.

**Table 4:** Effect of red osier dogwood extract on gut morphology of broiler chickens challenged intraperitoneally with *Salmonella Enteritidis* Lipopolysaccharide.

		Treatment^1^					Challenged model^2^			*P*-value		
Parameters	Challenged model^2^	Basal	BMD	0.3% ROD	0.5% ROD	SE Mean^3^	U	C	SE Mean^3^	Treatment effect	Model effect	Interaction effect
Duodenum												
Villus height (mm)	U	1.32	1.42	1.32	1.35	0.021	1.35	1.31	0.016	0.820	0.228	0.476
	C	1.33	1.30	1.33	1.29	0.025						
Villus width (mm)	U	0.17	0.15	0.15	0.16	0.007	0.16	0.15	0.004	0.684	0.487	0.192
	C	0.14	0.15	0.15	0.14	0.004						
Crypt depth (mm)	U	0.11	0.10	0.10	0.10	0.002	0.10	0.11	0.002	0.218	0.868	0.891
	C	0.12	0.10	0.10	0.10	0.003						
VH:CD^4^	U	13.14	15.98	13.42	14.08	0.550	14.15	13.23	0.334	0.224	0.152	0.767
	C	12.37	13.53	13.34	13.68	0.371						
Jejunum												
Villus height (mm)	U	0.72	0.76	0.72	0.75	0.017	0.73	0.71	0.013	0.874	0.476	0.441
	C	0.75	0.72	0.73	0.67	0.019						
Villus width (mm)	U	0.15	0.15	0.17	0.17	0.005	0.15	0.15	0.003	0.373	0.884	0.486
	C	0.16	0.16	0.16	0.16	0.005						
Crypt depth (mm)	U	0.07	0.08	0.06	0.06	0.003	0.06	0.06	0.002	0.102	0.486	0.797
	C	0.07	0.07	0.06	0.06	0.002						
VH:CD^4^	U	13.22	11.55	12.31	12.51	0.493	11.62	11.51	0.286	0.567	0.691	0.917
	C	12.36	11.46	12.44	11.68	0.292						
Ileum												
Villus height (mm)	U	0.36	0.35	0.36	0.38	0.010	0.36	0.38	0.008	0.460	0.151	0.939
	C	0.38	0.37	0.37	0.41	0.012						
Villus width (mm)	U	0.18	0.16	0.17	0.17	0.006	0.14	0.14	0.843	0.740	0.114	0.359
	C	0.15	0.16	0.16	0.14	1.690						
Crypt depth (mm)	U	0.09	0.09	0.08	0.10	0.003	0.09	0.09	0.002	0.006	0.443	0.208
	C	0.09 ^ab^	0.09 ^ab^	0.10 ^a^	0.11 ^a^	0.004						
VH:CD^4^	U	3.96 ^b^	3.95 ^b^	5.05 ^a^	3.92 ^b^	0.146	4.06	4.37	0.108	0.228	0.082	0.178
	C	4.62	4.40	4.54	4.61	0.158						

^ab^In a row, means assigned different lowercase letters are significantly different, *P* < 0.05 (Tukey's procedure).

1 Basal, negative control diet, BMD (bacitracin methylene disalicylate) antibiotic diet, 0.3% ROD, diet containing 0.3% red osier dogwood extract, 0.5% ROD, diet containing 0.5% red osier dogwood extract.

2 U = Unchallenged group; C = Challenged group.

3 SEM, standard error of the mean.

4 VH:CD, Villus height:crypt depth ratio.

### Serum Biochemistry

The effect of ROD extract on serum biochemical indices of broiler chickens challenged or unchallenged intraperitoneally with *SE*-LPS is shown in Table 5. No interaction was detected on the measured blood parameters. Similarly, no treatment effect was observed on the plasma biochemical indices of birds in the challenged group. Dietary Supplementation of 0.3% ROD extract significantly increased and reduced (*P* < 0.05) globulin (**GLB**) and albumin:globulin (**A:G**), respectively in the unchallenged birds compared with the antibiotic treatment; however, they were marginally similar to those receiving dietary 0.5% ROD extract and control treatments. With respect to the challenge model effects, calcium, iron, total protein (**TP**), cholesterol (**CHOL**), albumin (**ALB**), GLB, and gamma-glutamyl transferase (**GGT)** were significantly higher (*P* < 0.05) among the challenged birds, while lipase and creatine kinase (**CK**) were significantly higher (*P* < 0.05) among the unchallenged group.

**Table 5:** Effect of red osier dogwood extract on plasma biochemical indices of broiler chickens challenged intraperitoneally with *Salmonella Enteritidis* Lipopolysaccharide.

		Treatment^1^					Challenge model^2^			*P*-value		
Parameters	Challenge model^2^	Basal	BMD	0.3% ROD	0.5% ROD	SE Mean^3^	U	C	SE Mean^3^	Treatment effect	Model effect	Interaction effect
Calcium (mmol/L)	U	2.58	2.41	2.70	2.54	0.048	2.56 ^b^	2.71 ^a^	0.031	0.099	0.014	0.836
	C	2.71	2.64	2.78	2.69	0.037						
Phosphorus (mmol/L)	U	1.97	2.18	1.99	1.82	0.062	1.99	1.98	0.043	0.812	0.922	0.152
	C	1.89	1.92	2.01	2.11	0.061						
Magnesium (mmol/L)	U	0.75	0.80	0.73	0.75	0.023	0.74	0.77	0.016	0.875	0.263	0.228
	C	0.82	0.72	0.80	0.84	0.022						
Sodium (mmol/L)	U	140.0	141.0	141.0	140.0	1.290	140.0	142.0	0.884	0.772	0.404	0.912
	C	139.0	142.0	144.0	142.0	1.210						
Potassium (mmol/L)	U	6.83	6.81	6.76	6.48	0.220	6.72	7.07	0.154	0.953	0.685	0.536
	C	7.15	6.70	6.94	7.50	0.220						
Na:K^3^	U	20.88	22.35	20.91	21.74	0.814	21.47	20.75	0.491	0.977	0.878	0.510
	C	20.50	21.80	21.30	19.40	0.568						
Chloride (mmol/L)	U	104.0	108.0	107.0	106.0	0.920	106.0	105.0	0.622	0.725	0.778	0.885
	C	104.0	106.0	107.0	105.0	0.851						
Iron (umol/L)	U	17.90	17.80	15.89	18.20	0.519	17.45 ^b^	22.55 ^a^	0.520	0.091	<0.05	0.629
	C	22.20	21.80	21.10	25.10	0.699						
Amylase (U/L)	U	899.0	470.0	808.0	617.0	115.0	699.0	539.0	63.10	0.424	0.063	0.575
	C	556.0	519.0	574.0	509.0	47.40						
Lipase (U/L)	U	26.88	20.45	18.83	24.39	3.690	22.19 ^a^	19.43 ^b^	2.020	0.616	0.011	0.837
	C	21.40	19.00	17.80	19.50	1.350						
Bile acids (mmol/L)	U	17.78	19.61	22.17	19.70	0.934	19.82	21.88	0.662	0.188	0.502	0.679
	C	21.00	20.80	22.60	23.10	0.942						
Glucose (mmol/L)	U	13.38	12.85	13.64	13.34	0.366	13.30	13.89	0.212	0.694	0.739	0.729
	C	13.59	13.77	13.90	14.30	0.222						
T.Protein (g/L)^4^	U	22.82	21.54	25.28	21.81	0.574	23.19 ^b^	26.30 ^a^	0.463	0.079	<0.05	0.608
	C	27.00	24.50	27.00	26.70	0.639						
Cholesterol (mmol/L)	U	2.61	2.60	2.78	2.79	0.083	2.70 ^b^	3.05 ^a^	0.061	0.658	0.005	0.962
	C	3.04	2.96	3.03	3.16	0.082						
Uric Acid (umol/L)	U	393.0	298.0	321.0	326.0	16.10	335.0	358.0	10.30	0.334	0.724	0.131
	C	354.0	379.0	380.0	320.0	13.10						
Urea (mmol/L)	U	0.33	0.32	0.32	0.36	0.016	0.33	0.33	0.011	0.834	0.815	0.127
	C	0.33	0.35	0.36	0.27	0.015						
CK (U/L)^5^	U	2,629	3,246	2,393	2,024	311.0	2573 ^a^	2088 ^b^	179.0	0.218	0.003	0.714
	C	2,186	2,226	2,188	1,754	145.0						
Creatinine (umol/L)	U	0.00	0.00	0.00	0.00	0.136	0.00	0.00	0.118	0.387	0.112	-
	C	0.00	0.00	0.50	0.00	0.186						
Albumin (g/L)	U	9.32	9.12	9.27	9.03	0.193	9.22 ^b^	10.20 ^a^	0.151	0.908	0.002	0.769
	C	10.20	9.90	10.10	10.60	0.209						
Globulin (g/L)	U	13.42 ^ab^	12.37 ^b^	15.86 ^a^	12.73 ^ab^	0.468	13.78 ^b^	15.92 ^a^	0.377	0.035	0.003	0.521
	C	17.50	15.00	15.50	16.50	0.538						
A:G^6^	U	0.69 ^ab^	0.74 ^a^	0.59 ^b^	0.71 ^ab^	0.021	0.67	0.66	0.015	0.044	0.298	0.585
	C	0.62	0.70	0.64	0.67	0.022						
ALP (U/L)^7^	U	5619	5464	6598	6828	587.0	6127	7393	380.0	0.887	0.246	0.698
	C	7434	7682	7875	6582	484.0						
ALT (U/L)^8^	U	5.76	5.79	6.17	8.16	0.574	6.47	5.93	0.411	0.825	0.189	0.071
	C	6.50	7.80	5.10	4.30	0.583						
AST (U/L)^9^	U	169.9	180.6	174.1	170.5	4.110	174.0	172.0	2.540	0.571	0.900	0.746
	C	165.0	174.0	171.0	178.0	3.080						
GGT (U/L)^10^	U	8.85	10.13	9.70	9.79	0.428	9.43 ^b^	11.48 ^a^	0.438	0.633	0.005	0.536
	C	12.07	11.86	10.76	13.81	0.710						
T. Bilirubin (umol/L)^11^	U	0.00	0.00	0.00	0.00	0.066	0.00	0.00	0.059	0.908	0.831	-
	C	0.00	0.00	0.00	0.00	0.098	0.00	0.00				

1 Basal, negative control diet, BMD (bacitracin methylene disalicylate) antibiotic diet, 0.3% ROD, diet containing 0.3% red osier dogwood extract, 0.5% ROD, diet containing 0.5% red osier dogwood extract.

2 U = Unchallenged group; C = Challenged group.

3 Na:K, Sodium:Potassium ratio.

4 T. Protein, total protein.

5 CK, creatine kinase.

6 A:G, Albumin Globulin ratio.

7 ALP, alkaline phosphatase.

8 ALT, alanine aminotransferase.

9 AST, aspartate aminotransferase.

10 GGT, gamma-glutamyl transferase.

11 T.Bilirubin = total bilirubin. ^ab^In a row, means assigned different lowercase letters are significantly different, *P* < 0.05 (Tukey's procedure).

### Serum Immunoglobulins, Antioxidant Status, and Relative Weight of Immune Organs

The effect of ROD extract on serum immunoglobulin Y and M, antioxidant status, and relative weight of immune organs of broiler chickens challenged or unchallenged intraperitoneally with *SE*-LPS is shown in Table 6. No interaction was observed. Also, serum IgY and IgM, SOD, and TAP were not significantly affected (*P* > 0.05) by the dietary inclusion of 0.3 and 0.5% ROD extract. A significant model effect (*P* < 0.05) was observed on serum IgM and was seen to be higher among challenged birds compared to the unchallenged birds. The dietary treatments did not influence (*P* > 0.05) the relative weight of immune organs. However, the challenge model had a significant effect (*P* < 0.05) on relative spleen weight, which was higher among the challenged birds compared to the unchallenged ones. In addition, no interaction between the dietary treatment and challenged model was noticed.

**Table 6:** Effect of red osier dogwood extract on serum immunoglobulin Y and M, antioxidant status, and relative weight of immune organs of broiler chickens challenged intraperitoneally with *Salmonella Enteritidis* Lipopolysaccharide.

		Treatment^1^					Challenge model^2^			*P*-value		
Parameters	Challenge model^2^	Basal	BMD	0.3% ROD	0.5% ROD	SE Mean^3^	U	C	SE Mean^3^	Treatment effect	Model effect	Interaction effect
Serum IgY (mg/mL)	U	4.92	4.36	7.72	4.74	1.370	6.20	7.41	1.120	0.349	0.352	0.790
	C	6.27	6.41	8.71	8.60	1.780						
Serum IgM (mg/mL)	U	0.42	0.37	0.34	0.34	0.034	0.33^b^	0.43^a^	0.030	0.493	0.012	0.199
	C	0.49	0.35	0.54	0.58	0.047						
SOD (U/mL)^3^	U	1.61	1.79	1.62	1.59	0.050	1.62	1.59	0.041	0.409	0.232	-
	C	1.61	1.52	1.62	1.53	0.063						
TAP (uM copper reducing equivalents)^4^	U	1,750	1,638	1,642	1,787	47.50	1,705	1,739	31.50	0.823	0.601	0.630
	C	1,699	1,756	1,765	1,736	41.90						
Relative liver weight (% of BW of birds) 5	U	2.70	2.55	2.83	2.59	0.050	2.67	2.78	0.030	0.219	0.091	0.101
	C	2.84	2.66	2.69	2.91	0.040						
Relative spleen weight (% of BW of birds) 5	U	0.08	0.07	0.08	0.07	0.003	0.07 ^b^	0.09 ^a^	0.002	0.867	<0.005	0.151
	C	0.09	0.09	0.08	0.10	0.003						

^ab^In a row, means assigned different lowercase letters are significantly different, *P* < 0.05 (Tukey's procedure).

1 Basal, negative control diet, BMD (bacitracin methylene disalicylate) antibiotic diet, 0.3% ROD, diet containing 0.3% red osier dogwood extract, 0.5% ROD, diet containing 0.5% red osier dogwood extract.

2 U = Unchallenged group; C = Challenged group.

3 SOD, superoxide dismutase.

4 TAP, total antioxidant power.

5 Relative weight of liver or spleen = (weight of liver or spleen (in grams) × 100) / bodyweight of bird (in grams).

### Cecal Short-Chain Fatty Acid Concentration

The ceca SCFA concentration and total eubacteria counts of ROD-extract-fed-broiler chickens challenged or unchallenged intraperitoneally with *SE*-LPS is presented in Table 7. Compared to antibiotic and control treatments, dietary supplementation of 0.3 and 0.5% ROD extract did not affect (*P* > 0.05) total eubacteria count, short chain fatty acid (**SCFA**), acetic acid (**AA**), propionic acid (**PA**), butyric acid (**BA**), valeric acid (**VA**), lactic acid (**LA**), branched chain fatty acid (**BCFA**), and volatile (**VFA)**. In addition, no significant difference (*P* > 0.05) existed between challenge and unchallenged groups. No interaction was observed on the ceca short-chain fatty acid concentration; however, there was a significant interaction effect (*P* < 0.05) on the total eubacteria count.

**Table 7:** Effect of red osier dogwood extract on total eubacteria count and short-chain fatty acids concentration in the ceca of broiler chickens challenged with *Salmonella Enteritidis* Lipopolysaccharide.

		Treatment^1^					Challenge model^2^			*P*-value		
Parameters	Challenge model^2^	Basal	BMD	0.3% ROD	0.5% ROD	SE Mean^3^	U	C	SE Mean^3^	Treatment effect	Model effect	Interaction effect
Total eubacteria × 10^12^ (16S rRNA gene copies/gram of sample)	U	4.41	4.29	11.62	7.05	1.650	6.27	7.71	1.071	0.290	0.246	0.028
	C	11.63	10.81	8.56	5.86	1.380						
SCFA (mmol/kg)^3^	U	37.09	65.04	92.63	88.04	19.20	66.6	74.59	11.00	0.152	0.535	0.241
	C	76.03	75.32	67.74	86.10	10.70						
Acetic acid (mmol/kg)	U	26.16	46.60	70.04	65.02	13.40	48.54	53.1	7.840	0.104	0.608	0.163
	C	56.67	49.11	48.16	90.62	8.020						
Propionic acid (mmol/kg)	U	1.86	3.20	1.80	2.07	0.856	2.63	3.37	0.475	0.551	0.135	-
	C	3.95	3.13	2.54	4.74	0.410						
Butyric acid (mmol/kg)	U	10.29	12.36	18.64	16.08	4.230	14.31	15.16	2.350	0.445	0.796	0.716
	C	16.87	13.13	14.95	23.39	1.970						
Valeric acid (mmol/kg)	U	0.82	0.77	1.04	0.90	0.183	0.89	0.79	0.122	0.314	0.701	-
	C	0.88	0.64	0.59	0.85	0.162						
Lactic acid (mmol/kg)	U	0.00	0.00	0.23	0.00	0.979	0.00	0.06	0.547	0.057	0.323	-
	C	0.00	0.00	0.70	1.01	0.481						
BCFAs (mmol/kg)^4^	U	0.29	0.74	0.44	0.54	0.108	0.43	0.43	0.133	0.667	0.460	-
	C	1.20	0.36	0.94	1.39	0.244						
VFAs (mmol/kg)^5^	U	36.65	65.04	91.26	86.48	18.60	65.86	73.33	10.70	0.167	0.557	0.222
	C	79.72	66.35	67.19	121.6	10.40						

1 Basal, negative control diet, BMD (bacitracin methylene disalicylate) antibiotic diet, 0.3% ROD, diet containing 0.3% red osier dogwood extract, 0.5% ROD, diet containing 0.5% red osier dogwood extract.

2 U = Unchallenged group; C = Challenged group.

3 SCFA, Short chain fatty acid.

4 BCFA, Branch chain fatty acid.

5 VFA, Volatile fatty acid.

### Cecal Microbiota

**Figure 3:**
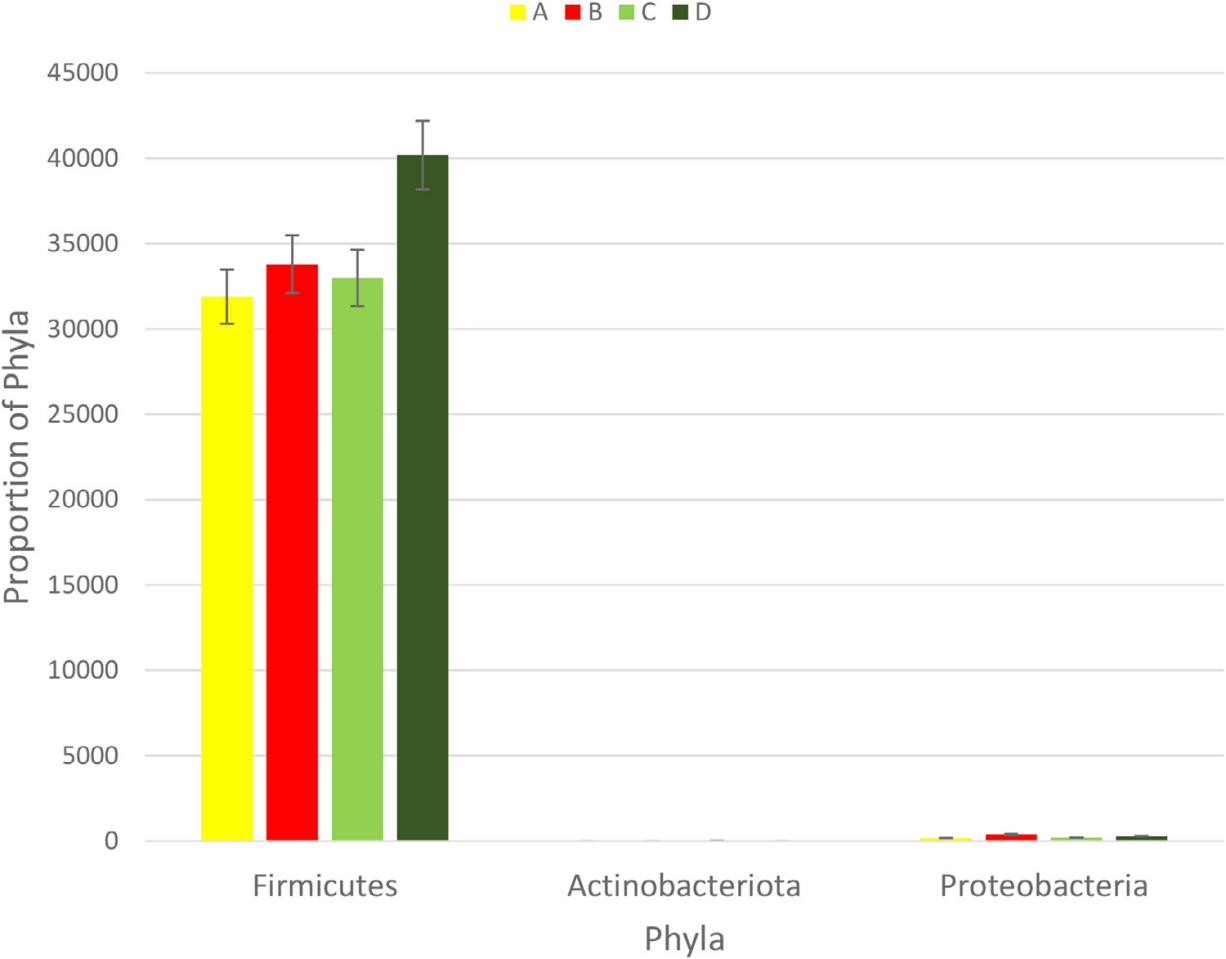
Proportion of the most abundance bacteria phyla in the ceca of broiler chickens challenged intraperitoneally with or without *SE*-LPS and fed red osier dogwood extract as a substitute for in-feed antibiotics. Treatment: A = Negative control, B = Antibiotic (bacitracin methylene disalicylate) diet, C = diet containing 0.3% red osier dogwood extract, and D = diet containing 0.5% red osier dogwood extract.

**Figure 4:**
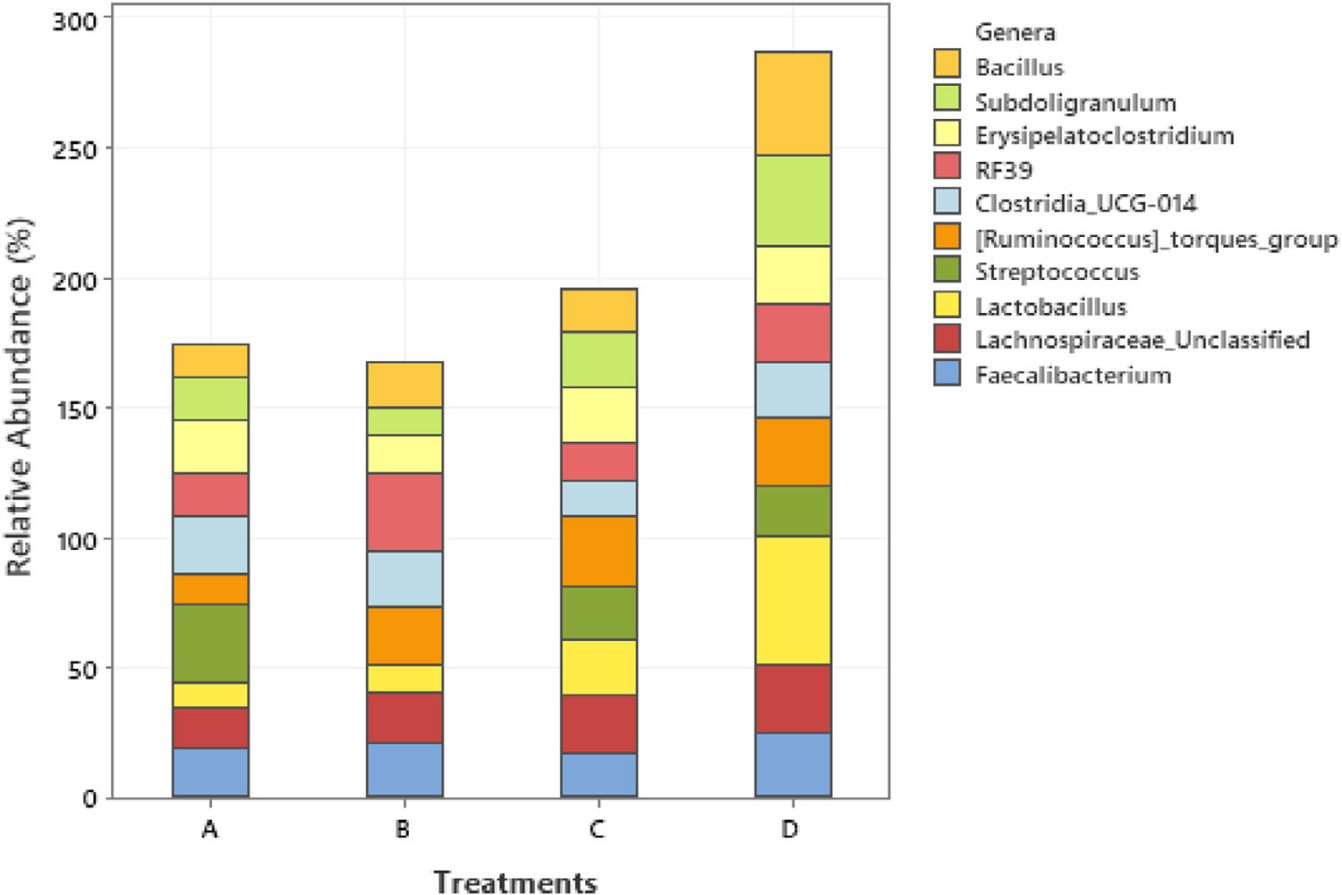
Percentage relative abundance of the most abundant bacteria genera in the ceca of broiler chickens challenged intraperitoneally with or without *SE*-LPS and fed 4 different dietary treatments. Treatment: A = Negative control, B = Antibiotic (bacitracin methylene disalicylate) diet, C = diet containing 0.3% red osier dogwood extract, and D = diet containing 0.5% red osier dogwood extract.

**Figure 5:**
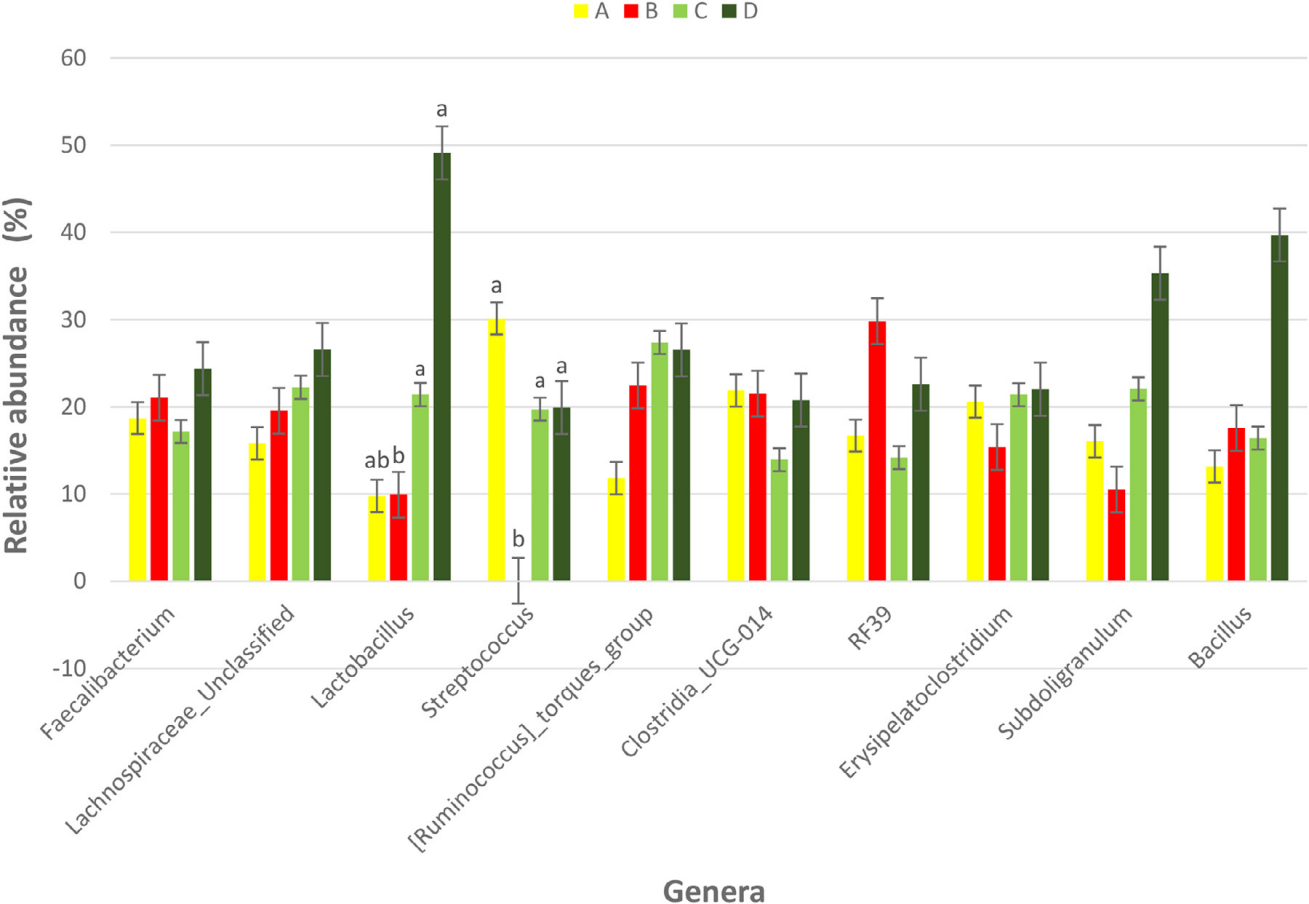
Percentage relative abundance of the top 10 most abundant bacteria genera in the ceca of broiler chickens challenged intraperitoneally with or without *SE*-LPS and fed 4 different dietary treatments. Treatment: A = Negative control, B = Antibiotic (bacitracin methylene disalicylate) diet, C = diet containing 0.3% red osier dogwood extract, and D = diet containing 0.5% red osier dogwood extract. Note: Genera without a mean separation have their *P*-value greater than 0.05.

**Figure 6:**
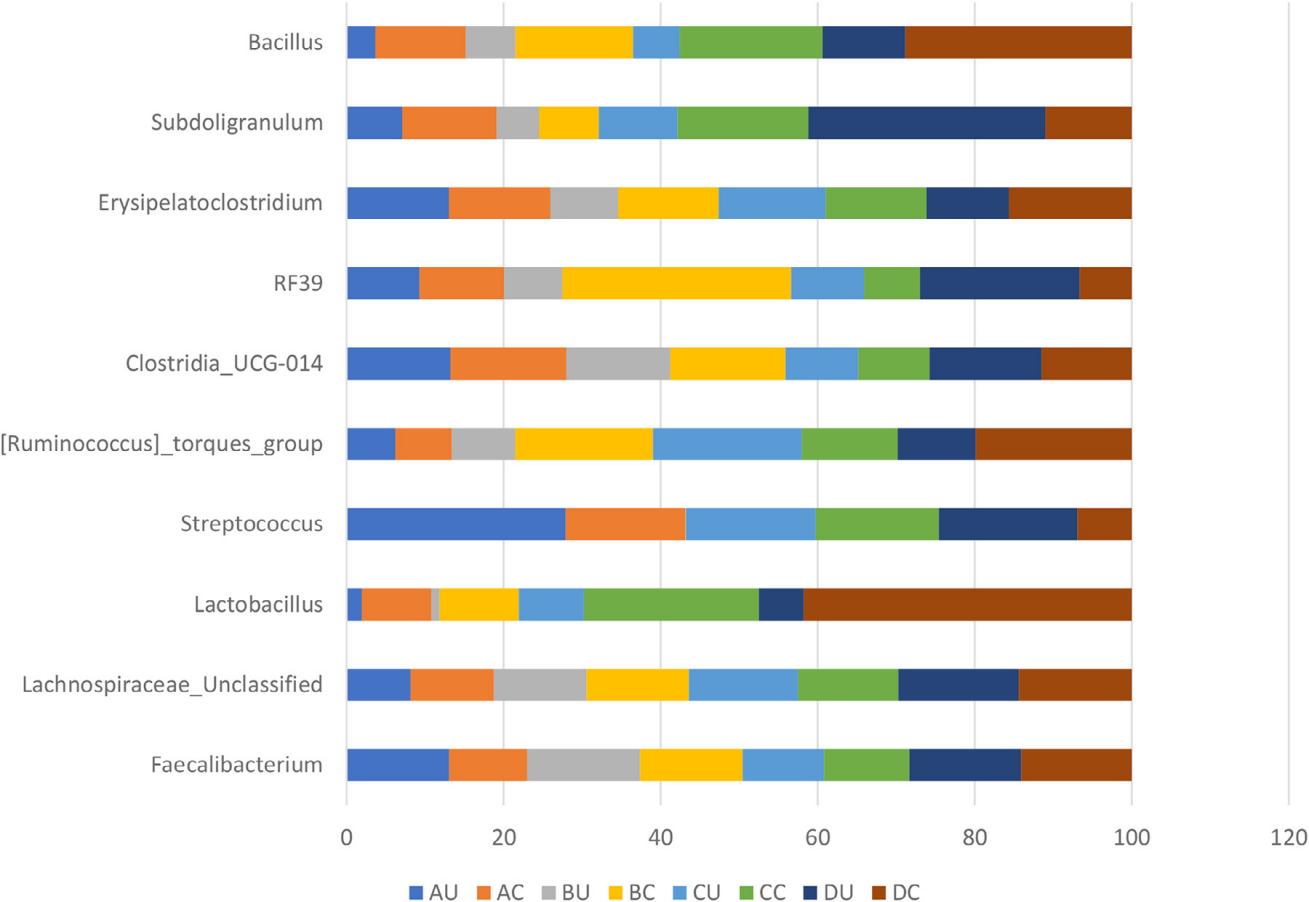
Percentage relative abundance of the top 10 most abundant bacteria genera in the ceca of broiler chickens challenged intraperitoneally with or without *SE*-LPS and fed 4 different dietary treatments. Treatment: A = Negative control, B = Antibiotic (bacitracin methylene disalicylate) diet, C = diet containing 0.3% red osier dogwood extract, and D = diet containing 0.5% red osier dogwood extract. Challenge groups: U = group of birds that were not challenged with *SE*-LPS, C = group of birds that were challenged with SE-LPS.

**Figure 7:**
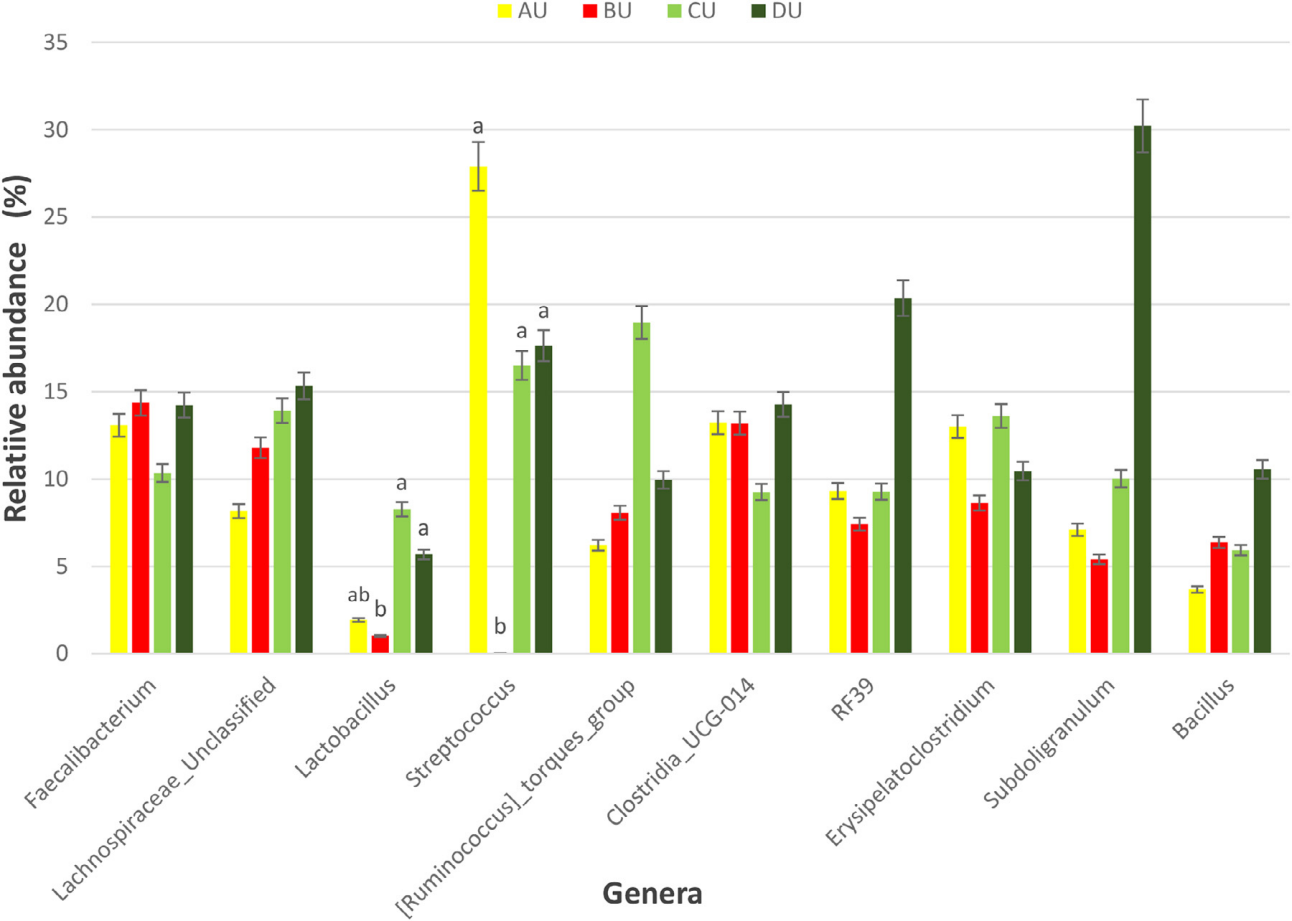
Percentage relative abundance of the top 10 most abundant bacteria genera in the ceca of *SE*-LPS-unchallenged broiler chickens fed 4 different dietary treatments. Treatment: A = Negative control, B = Antibiotic (bacitracin methylene disalicylate) diet, C = diet containing 0.3% red osier dogwood extract, and D = diet containing 0.5% red osier dogwood extract. Challenge groups: U = group of birds that were not challenged with *SE*-LPS, C = group of birds that were challenged with *SE*-LPS. Note: Genera without a mean separation have their *P*-value greater than 0.05.

**Figure 8:**
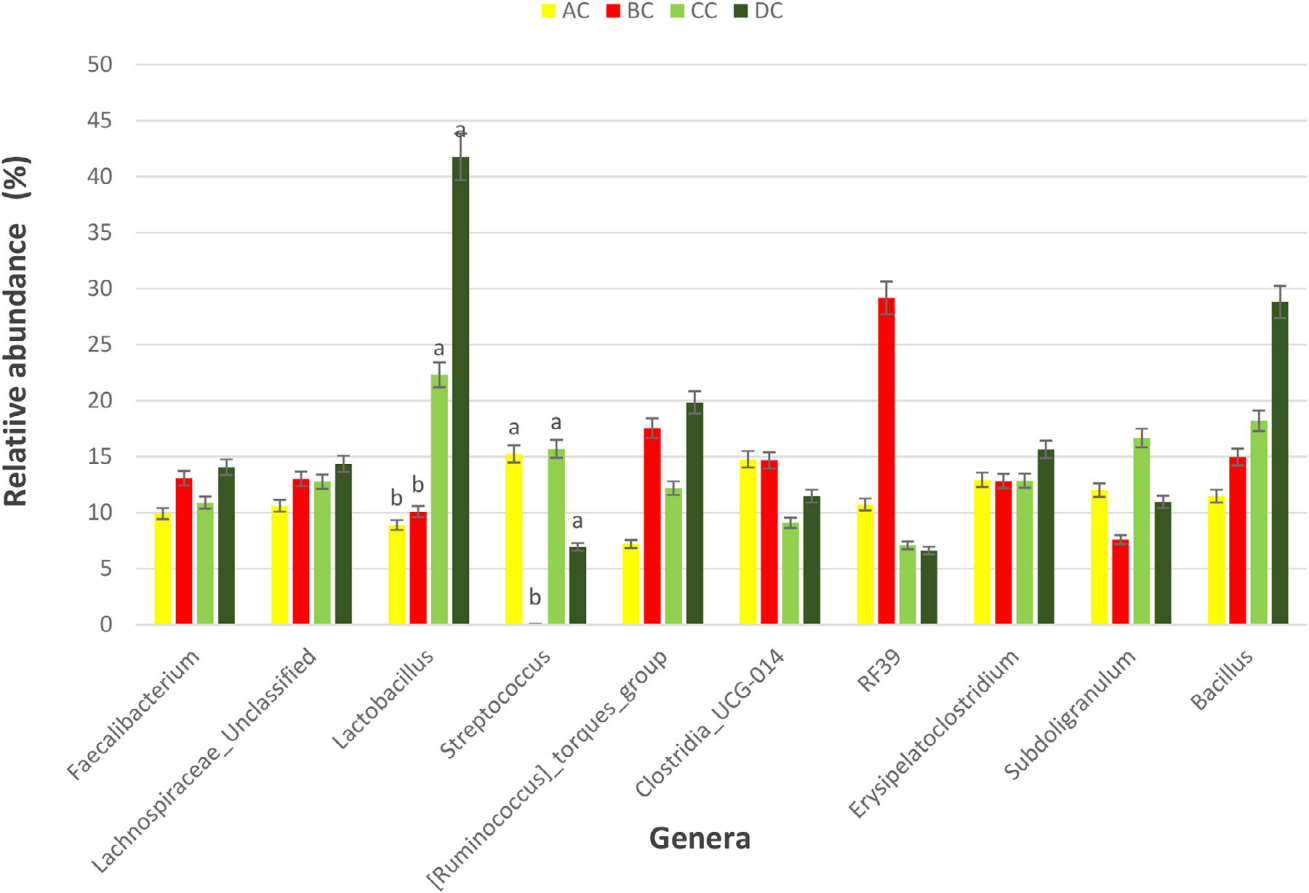
Percentage relative abundance of the top 10 most abundant bacteria genera in the ceca of *SE*-LPS-challenged broiler chickens fed 4 different dietary treatments. Treatment: A = Negative control, B = Antibiotic (bacitracin methylene disalicylate) diet, C = diet containing 0.3% red osier dogwood extract, and D = diet containing 0.5% red osier dogwood extract. Challenge groups: U = group of birds that were not challenged with *SE*-LPS, C = group of birds that were challenged with *SE*-LPS. Note: Genera without a mean separation have their *P*-value greater than 0.05.

**Figure 9:**
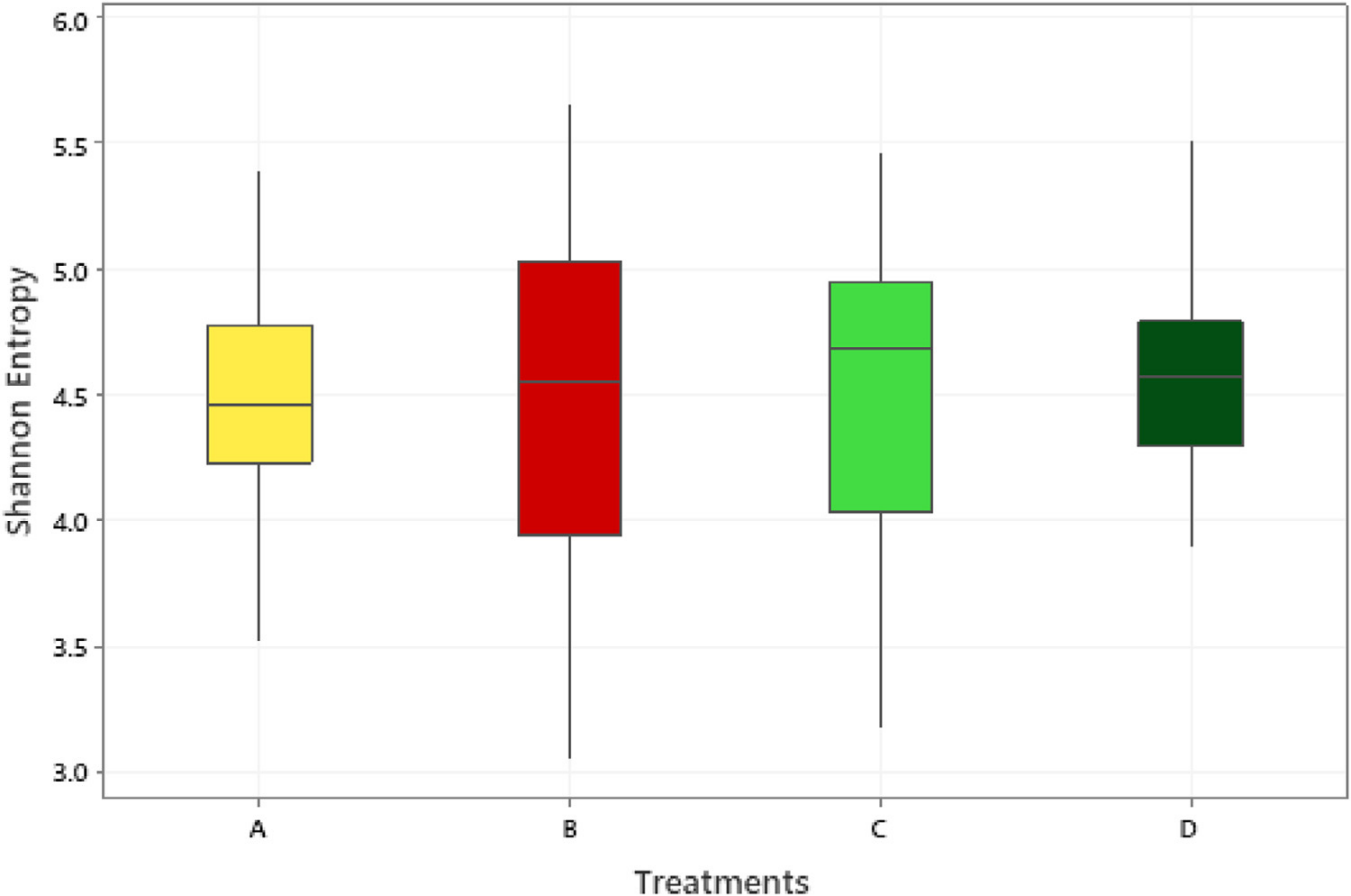
Box-and-whisker plot showing nonsignificant differences in the Shannon entropy (Alpha diversity) (*P* > 0.05). Ceca content was collected from 21-day-old broiler chickens fed four different dietary treatments. Treatment: A = Negative control, B = Antibiotic (bacitracin methylene disalicylate) diet, C = diet containing 0.3% red osier dogwood extract, and D = diet containing 0.5% red osier dogwood extract.

**Figure 10:**
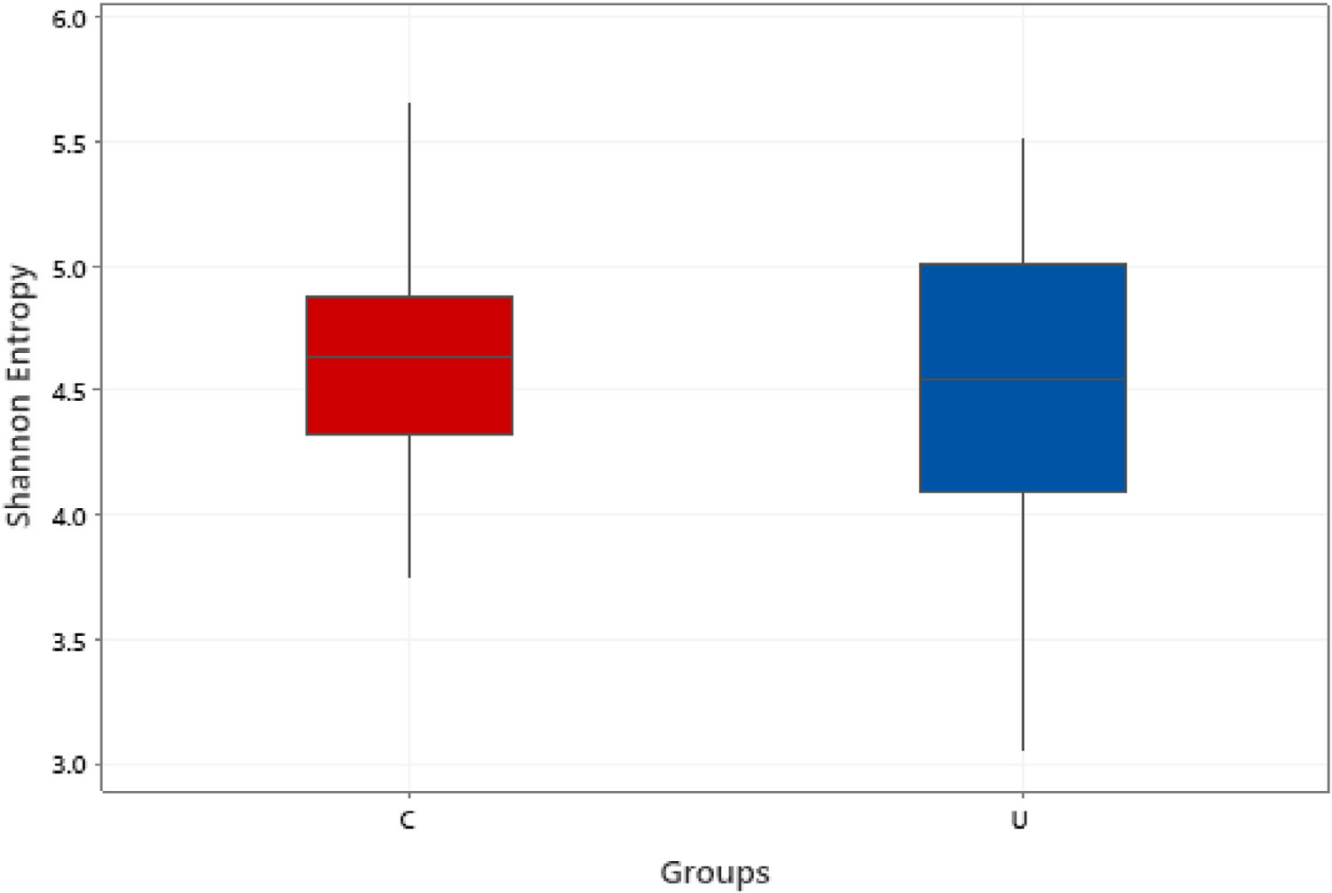
Box-and-whisker plot showing nonsignificant differences in the Shannon entropy (Alpha diversity) (*P* > 0.05). Ceca content was collected from 21-day-old broiler chickens challenged with *SE*-LPS and fed four different dietary treatments. Treatment: A = Negative control, B = Antibiotic (bacitracin methylene disalicylate) diet, C = diet containing 0.3% red osier dogwood extract, and D = diet containing 0.5% red osier dogwood extract.

**Figure 11:**
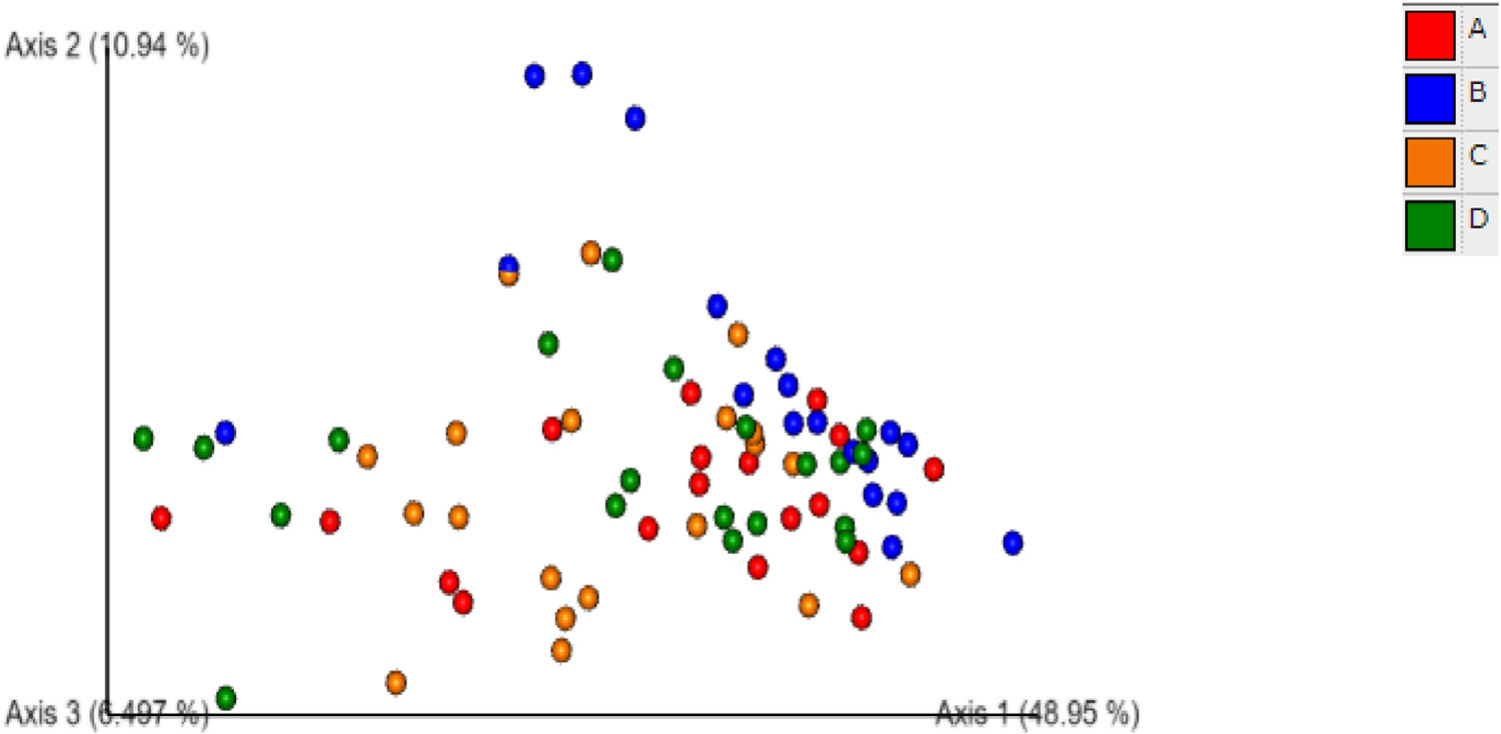
Multivariance analysis determined differences in beta-diversity among treatments. Treatment groups: A = Negative control, B = Antibiotic (bacitracin methylene disalicylate) diet, C = diet containing 0.3% red osier dogwood extract, and D = diet containing 0.5% red osier dogwood extract.

The effect of 0.3 and 0.5% ROD extract on the cecal microbiota of broiler chickens challenged or unchallenged intraperitoneally with *SE*-LPS is shown Figure 3, Figure 4, Figure 5, Figure 6, Figure 7, Figure 8, Figure 9, Figure 10, Figure 11, and Supplementary Figures 12 and 13. The aggregation of OTU into each taxonomic rank, as well as, the relative abundance of the most abundant phyla, and genera based on treatments, group, and treatment/group effects are presented in Figure 3, Figure 4, Figure 5, respectively. The percentage relative abundance of the three phyla namely, Actinobacteriota, Proteobacteria, and Firmicutes was not influenced by the dietary treatments; however, Firmicutes was the most abundant phylum. Unlike other genera, supplementation of 0.3 and 0.5% ROD extract significantly increased (*P* < 0.05) the percentage relative abundance of genera *Lactobacillus* and *Streptococcus* compared to the antibiotic treatments; however, they were similar to the control treatment regardless of the *SE*-LPS challenge (Figure 6–8). Furthermore, the Shannon diversity (i.e., specie richness) was not affected either by the dietary treatments or the challenge, as shown in Figures 9 and 10. In addition, a principal coordinate analysis showed a significant difference (*P* < 0.05) in the beta diversity in the cecal microbiota with more diversity observed among the birds fed 0.3%, 0.5% ROD extract, and control treatment as shown in Figure 11. There was no difference in the alpha and beta diversity between the challenged and unchallenged groups, as presented in Supplementary Figures 12 and 13.

## DISCUSSION

Phenolic compounds are known for their array of beneficial bioactivities, including antioxidant and selective antimicrobial capacities. Red osier dogwood extracts have been reported to contain a high amount of phenolic compounds of about 220 mg gallic acid equivalents g^−1^ dry weight (Isaak et al., 2013). The TPC in the ROD extract used in the current study was 238.81 mg GAE/g with gallic acid and quercetin being the most prevalent phenolic compounds. The combination of gallic acid and quercetin has commendable health benefits. Both polyphenols have been reported as a potential treatment for colorectal cancer in Wistar rats, as well as, causing an upregulation of bodily antioxidant enzymes in bleomycin-induced pulmonary fibrosis in rats (Mehrzadi et al., 2020; Patil and Killedar, 2021). In our study, the TPC of diets was observed to increase with an increasing amount of ROD extract inclusion.

In all of the growth response variables evaluated, no interactions were observed; thus, the results presented are discussed with respect to the main effects. The use of antibiotics for the acceleration of growth, improved feed conversion efficiency, and treating diseases has been affirmed and reaffirmed in literature (Sarmah et al., 2006; Mehdi et al., 2018; Shang et al., 2020). In the current study, AFI, AWG, FCR, and mortality were similar among broiler chickens fed dietary 0.3 and 0.5% ROD extract on d 7 compared to the antibiotic and control birds. This is similar to the findings of Mogire et al. (2021) who reported that the dietary inclusion of 0.1 and 0.3% ROD extract did not influence the growth performance of broiler chickens but were favorable to birds fed avilamycin diet. Furthermore, throughout the entire experimental period, no treatment effect was observed on the growth response of broiler chickens challenged with *SE*-LPS. Despite the higher concentration of *SE*-LPS injected intraperitoneally at 2 mg/kg, Shang et al. (2015) reported a similar growth performance of broiler chickens even in the presence of dietary antibiotics. According to Xie et al. (2000), clinical signs, including reduced feed intake, water intake, body weight, etc., were associated with broiler chickens receiving 5 mg of *Salmonella Typhimurium Thyphimurium* LPS per kg of BW. In addition, Rauber et al. (2014) and Guaiume (2005) also obtained a similar growth performance in broiler chickens injected intraperitoneally with 200 or 400 µg *E. coli* LPS. This suggests that the dose of *SE*-LPS used in the current study is within the maximum tolerable limit. Unlike Mogire et al. (2021), on d 14, 21, and at the overall basis, both 0.3 and 0.5% inclusion levels of ROD extract were found to marginally improve AWG of unchallenged birds compared to the same group of birds receiving antibiotics. This is attributable to the impact of the gallic acid and quercetin which are richly available in ROD extract. Samuel et al. (2017) and Zhang and Kim (2020) reported that gallic acid and quercetin, respectively, enhanced the growth performance of broiler chickens. The challenge model significantly affected AWG on d 14 and FCR on d 21 and was found to be improved among the unchallenged birds compared to the challenged group. This is in line with the findings of Yang et al. (2008) and Hu et al. (2011), where reduced daily gain was reported in broiler chickens exposed to 1 mg/kg *SE*-LPS and 0.5 mg/kg *E.coli* LPS, respectively.

There was neither dietary treatment nor challenge model effect on the duodenal and jejunal VH, VW, CD, and VH:CD and ileal VH and VW. The nonsignificant effect of ROD extract on the duodenal morphology is in line with the findings of Mogire et al. (2021). However, unlike our findings, Mogire et al. (2021) reported that 0.1 and 0.3% ROD extracts significantly increased VH:CD in the jejunum compared to the birds receiving antibiotics. In contrast to the antibiotic and control treatments, dietary supplementation of both 0.3 and 0.5% ROD extract significantly deepened ileal CD in the challenged group of birds, whereas ileal VH:CD was significantly highest among the unchallenged birds fed 0.3% ROD extract. In nursery pigs challenged with *E. coli* K88+, Jayaraman et al. (2018) reported that ROD extract supplementation increased VH:CD and reduced CD in the ileum. The inconsistent impact of ROD extract on gut morphology might be due to the absence or presence of different challenge models, animal species, and ROD inclusion rates as reported in most literature. The population and diversity of gut microbiota have been reported to increase down the GIT (Ursell et al., 2012), suggesting a higher abundance of bacteria population in the ileum compared to the duodenum and jejunum. An increase in VH and a decrease in CD are considered as desirable indicators for large surface area for absorption and improved gut morphology; however, deeper CD could also be considered a desirable trait as it permits renewal of the villus epithelia in response to inflammation caused by the pathogens (Yason et al., 1987; Adeleye et al., 2018) or their metabolites. This suggests that the increased ileal CD among the *SE*-LPS challenged broiler chickens receiving ROD extracts could be a result of the ameliorative mechanism of ROD extract in rejuvenating the ileal gut architecture.

Blood contains important biomarkers that could be used in the assessment of physiological and health status of animals. It is notable that information on the effects of dietary ROD extract on the blood biochemistry of broiler chickens does not exist in the literature. Plasma proteins, including ALB and GLB, are produced in the liver and perform complex physiological roles. A decrease in plasma ALB is purportedly associated with the incidence of malnutrition and renal impairment; while an increase in GLB level is related to chronic inflammation (Li et al., 2018). In the current study, GLB and A:G were higher and lower among the unchallenged birds that consumed 0.3% ROD extract compared to the antibiotic-treated birds. The effect of plant extracts on the blood parameters of broiler chickens is sometimes controversial in the literature. In some studies, plant extracts were reported to reduce serum GLB (Soltan et al., 2008) and increase ALB and A:G in infection-free broiler chickens (Soltan et al., 2008; Sharma et al., 2015). In contrast, an increase in serum GLB concentration was also reported in unchallenged broiler chickens fed dietary plant extract (Ismail et al., 2020). Since the 0.3 and 0.5% ROD extract did not negatively affect liver enzymes, growth parameters, and gut morphology compared to the antibiotic and control treatments, the reduced ALB and increased GLB cannot be associated with malnutrition or chronic inflammation in birds. Besides this, the GLB reported in our study is within a normal range of 5 to 18.0 g/L reported by Thrall (2007). This suggests that ROD extract supplementation did not adversely affect the plasma biochemical indices of broiler chickens. Comparing the challenge model, calcium, iron, TP, CHOL, ALB, GLB, and GGT were significantly higher among the challenged birds, while lipase and CK were significantly higher among the unchallenged group. Elevated calcium, iron, TP, CHOL, GLB, GGT, lipase, and CK have been associated with immune-related diseases, and kidney or intestinal disease in animals (Williams et al., 2021). While calcium ions are known to play a key role in the regulation of the circulatory system and cell-to-cell communication, their increased accumulation is noteworthily associated with hemolytic anemia diseases including sickle cell, ß-thalassemia, and familial phosphofructokinase deficiency (Stafford et al. 2017). This further suggests that elevated calcium ions in the body could impair glycolytic ATP formation – an essentially important cellular energy in the body. Xie et al. (2000) demonstrated that total plasma protein concentration increases between 24 and 48 h after LPS challenge. Furthermore, in support of our findings, Sharma et al. (2015) reported an increase in GLB when broiler chickens were challenged with *E. coli*.

There were no interaction effect and challenge model effect on TAP and SOD. Similar to the antibiotic and control treatments, dietary supplementation of ROD extract did not influence the serum TAP and SOD of broiler chickens challenged or unchallenged with *SE*-LPS. Antimicrobial growth promoters have been used to improve antioxidant status of weaned pigs (Koo et al., 2020). The result obtained in the current study suggests that supplementation of ROD extract maintained TAP and SOD in the same capacity of antibiotics. Contrary to our findings, there was a significant increase in the serum SOD fed 4% ROD plant product compared to weaned piglets fed antibiotics (Amarakoon, 2017; Koo et al., 2020). This could be due to the difference in the ROD plant product and its higher inclusion level at 4% used in the studies. Furthermore, ROD supplementation at 0.3 and 0.5% did not affect serum IgY and IgM of broiler chickens; however, IgM was significantly higher among the challenged group of birds compared to the unchallenged. According to Larsson et al. (1993) and Rathnapraba et al. (2007), serum IgM is the first antibody produced during the first week postinfection. Thus, the higher IgM among the *SE*-LPS challenged chickens is not unexpected given the presence of *SE*-LPS – an immune stressor.

The gut microbiota performs an indispensable role in influencing the health and performance of poultry birds. The gut microbiota of poultry is mostly reported to be dominated by bacteria species from the phylum Firmicutes, Bacteroidetes, Actinobacteria, Fusobacteria, Proteobacteria (Ali et al., 2021), and Verrucomicrobia; however, Firmicutes and Bacteroidetes are the largest phyla (Qin et al., 2010; Almeida et al., 2019; Forster et al., 2019). Given the novelty of the ROD extract, their impact on the intestinal microbiota of broiler chickens is very scanty. Regardless of the challenge model, the cecal microbiota of broiler chickens fed the dietary treatments did not significantly influence the bacteria phyla namely, Firmicutes, Actinobacteria, and Proteobacteria and were dominated by Firmicutes. It is surprising that Bacteroidetes were not detected in the cecal content of broiler chickens in our study. However, gut microbiota studies with no Bacteroidetes have been reported in literature, particularly with the use of broad-spectrum antibiotics (Carvalho et al., 2012; Dubourg et al., 2013; Stanley et al., 2013; Oladokun et al., 2021). The hypervariable region V4–V5 targeted using the 16S rRNA gene sequencing could be responsible for the absence of phylum Bacteroidetes in our study. Bukin et al. (2019) demonstrated that hypervariable regions play a significant role in the precision and resolution for taxa, particularly among genera and species, with V2–V3 reported to have the highest resolution. In another study by García-López et al. (2020), a more diverse gut microbiota is detectable using V3–V4 hypervariable region. The hypervariable region V4–V5 region has reportedly been used for a wider microbial domain, including archaeal and bacteria domains (Fadeev et al., 2021). For future studies, we would recommend either V2–V3 or V3–V4 for chicken's gut microbiota analysis because they are more specific for bacteria alone. With or without the *SE*-LPS challenge, 0.3 and 0.5% ROD extract conferred a more beneficial effect by significantly increasing the abundance of genera *Lactobacillus* and *Streptococcus* compared to the antibiotic treatment. In contrast to antibiotic use, plant materials rich in polyphenols have been consistently shown to increase the population of gut-friendly *Lactobacillus* spp (Giannenas et al., 2018; Abolfathi et al., 2019; Erinle et al., 2022). Crisol-Martínez et al. (2017) reported that the use of bacitracin diminishes the abundance of Lactobacilli in the gut. Lactobacillus play their beneficial role in the gut by producing lactate, maintaining intestinal barrier function, particularly in immune-related diseased conditions, and regulating the expression of heat shock proteins and tight junction proteins (Liu et al., 2015; Honda and Littman, 2016). Like most plant extracts, ROD extract exerted a better gut improvement influence than antibiotics. Although, ROD extract increased Streptococcus in the chickens, compared to antibiotic treatment, however, it was similar to the control-fed birds. Some *Streptococcus* spp are known for their pathogenic virulence; however, many streptococcal species including *S. salivarius, S. dentisani, S. oligofermentans*, and *S*. A12, have been reported to possess antimicrobial properties by producing bacteriocins, proteases, or hydrogen peroxide (Huang et al., 2016; Llena et al., 2019; Ferrer et al., 2020). This suggests that the higher abundance of genus Streptococcus does not always imply opportunistic pathogens. Dietary supplementation of 0.3 and 0.5% ROD extract did not influence species richness and diversity in the gut ecosystem. The dietary treatments had significantly different beta diversity of the microbial population. This could be explained by the consistently higher abundance of Lactobacillus and Streptococcus in the ROD extracts and control treatment compared to the antibiotic treatment. Orlewska et al. (2018a,b) reported an altered diversity in soil microbial communities following antibiotic application. Despite the significant effect of ROD extract on the cecal microbiota, there was no corresponding effect on the SCFA profile at the cecum. This is not unexpected as the number of total eubacteria was not altered by the dietary treatments and *SE*-LPS challenge. Erinle et al. (2022) speculated that uniform copies of total eubacteria in cecal content of birds often give rise to unaltered cecal SCFA concentrations. However, there was a significant interaction effect between treatment and challenge model on total eubacteria, which did not, in turn, influence the concentration of SCFA profile of the birds.

The relative weight of immune organs was not affected by the dietary supplementation of ROD extract. This corroborates the report of Mogire et al. (2021), where there was no difference in the relative weight of liver and spleen of broiler chickens fed either ROD extract, antibiotics or control diets. The spleen is one of the most critical immune organs in poultry species. Immune cells in the spleen were reported to help in the fight against pathogenic microbes through specific immune response mechanisms (Dailey, 2002). In another study involving 500 µg/mL *Salmonella Typhimurium* LPS challenge, (Rauber et al., 2014) reported that the relative weight of liver remained unaffected. Comparing unchallenged versus challenged groups, the relative spleen weight was observed to be significantly higher among birds in the latter compared to the former. According to Ahiwe et al. (2019), increased spleen weight was reported in broiler chickens challenged with *Salmonella Typhimurium* LPS. In the presence of LPS antigen, there is a high propensity of hyperplasia, which causes inflammation by activating inflammatory cells. An increase in the size of immune organs could be associated with increased immune activities to counteract the effect of stressors, including pathogens or their metabolites. Thus, the increased spleen size among the challenged group of birds could be a part of the birds’ innate defense mechanism against the *SE*-LPS.

## CONCLUSIONS

Based on the results obtained, the *SE*-LPS depressed AWG and FCR during d 14 and 21, respectively. However, dietary supplementation of ROD extract at 0.3 and 0.5% maintained the growth performance of broiler chicken throughout the production phase in the equal capacity of the antibiotic, regardless of the *SE-*LPS challenge. Additionally, CD and VH:CD of the birds were improved in the ileum when both levels of ROD extract were supplemented into broiler chicken's diets compared to the antibiotics-fed birds, however, it was best at the 0.3% inclusion level. Furthermore, dietary supplementation of 0.3 and 0.5% ROD extract increased the abundance of Lactobacillus genera while not compromising blood biochemical indices, cecal SCFA concentrations, and innate antioxidant and immune status of the birds. This study, therefore, suggests that dietary supplementation of ROD extract at 0.3 or 0.5% could be a potential consideration for replacing antibiotics in broiler chicken nutrition.

## Appendix Supplementary materials

Image, application 1
