# Supplementary material for: Effect of red osier dogwood extract on growth performance, blood biochemical parameters, and gut functionality of broiler chickens challenged or unchallenged intraperitoneally with Salmonella Enteritidis lipopolysaccharide
Source: Poult Sci. 2022 Mar 18;101(7):101861. doi: 10.1016/j.psj.2022.101861 (PMC9118149; doi:10.1016/j.psj.2022.101861)
Supplement: Supplementary file 1 [file mmc1.docx]

**Supplementary Figures**

| 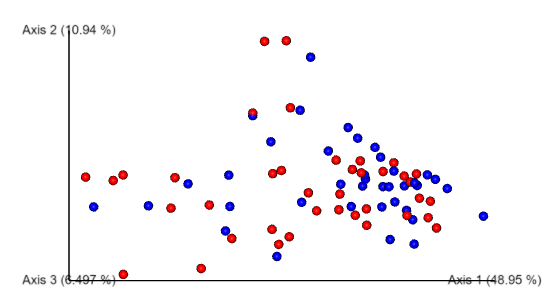 | 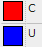 |
| --- | --- |

**Figure 12.** Multivariance analysis determined differences in beta-diversity between the challenge groups. Challenge groups: U = group of birds that were not challenged with *SE*-LPS, C = group of birds that were challenged with *SE*-LPS.

| 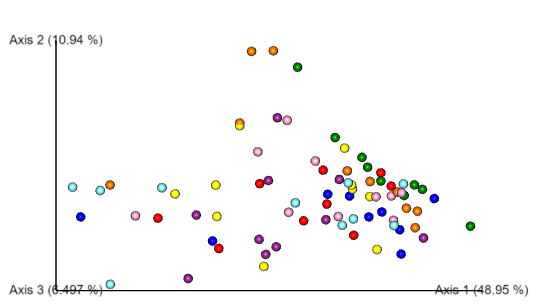 | 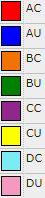 |
| --- | --- |

**Figure 13.** Multivariance analysis determined differences in beta-diversity among treatments and groups. Treatment groups: A = Negative control, B = Antibiotic (bacitracin methylene disalicylate) diet, C = diet containing 0.3% red osier dogwood extract, and D = diet containing 0.5% red osier dogwood extract. Challenge groups: U = group of birds that were not challenged with *SE*-LPS, C = group of birds that were challenged with *SE*-LPS
